# Supplementary material for: Genomic disturbance of vitellogenin 2 (vtg2) leads to vitellin membrane deficiencies and significant mortalities at early stages of embryonic development in zebrafish (Danio rerio)
Source: Sci Rep. 2023 Nov 1;13:18795. doi: 10.1038/s41598-023-46148-2 (PMC10620220; doi:10.1038/s41598-023-46148-2)
Supplement: Supplementary file 1 — Supplementary Information 1. [file 41598_2023_46148_MOESM1_ESM.pdf]

S1 Fig. A)Position of target sites and screening primers on *vtg2* gDNA. Introns are indicated in cyan, exones are indicated in plain text, screening primers are indicated in underlined bold text, target sites are indicated in magenta highlighted text along with corresponding labels to the right of the page, respectively. 21Fw, target1 forward primer; 21Rv, target1 reverse primer; 22Fw, target2 forward primer; 22Rv, target2 reverse primer; 23Fw, target3 forward primer; 23Rv, target3 reverse primer. sg21, sg22, and sg23: single guide RNAs (sgRNAs) for target sites 1, 2, and 3 for *vtg2*, respectively. Introduced mutation is indicated by strikethrough text.

>*vtg2*(gDNA)

ACAAACCACCAGCCATGAGAGCTGTTGTGCTTGCCTTGACTCTAGCCTTAGTGGGTAAGT
CAGATGTTTGAAAGCTCAATGTTTGTCAAATTTTATTTAGATGCAAATCTATTTTGAAGA
TTTTTTTCTCATTGCTATGTTTGTTTCTTTTAGCGAGTCAACAGAACAAACTTGGTAAGT
TTCGTTTTTTGGTTATCTTTTAAGAAGTTAATTTTACCTACAACACAAGAAAATCTAAAA
TATATACTTAAAAAAAAACATATGTTTCAGTTCCTGAGTTTGCCCATGATAAGACCTATG
TGTACAAGTATGAGGCTCTACTCTTGAGTGGTATTGTTCAAGAAGGACTGGCCAAAGCAG
GTATTCAAATCAAGAGCAAGGTTCTCCTCAGTGCCGCAACAGAGAATACCTTCCTGTTGA
AGGTAATGACTAATAAAACATGCCAGATCTTAGAAAAAAAACACAACCAAT**ACATTTCCC** **21 Fw**
AACGAGGGTGTATTACAGAAAATGTTACAATCAATTTAATTGAAAATGTCCCTTACAGTT
TGTGGATCCTCTTTTCTACGAGTATGCTGGCACTTGGCCCAAGGATCAAGTTTTCCTGC **sg21**
CACTAAGCTCAACTCAGCACTGGCTGCTCAGCTTCAGACTCCCATCAAGTTTGAGTACGC
TAATGGTGTGGTTGGCAAGGTTTTTGCACCAGCAGGAGTCTCTCCCTCAGTCTTGAACTT
GTACAGAGGTATCCTCAACATCCTTCAGCTTAACTTTAAGAAGACCCAGAACATCTACGA
GCTGCAAGAGGTAAAGTATCTTCCTAGTACATTTTTGATCTATATTTATCTTAGGTTTTA
CAATCTTCCTTCTAACATTATGACTTG**TTCTTCAAGGCTGGAGCTCA**GGGAGTTTGCAAA **21 Rv**
ACTCAGTATGTCATCAGTGAGGATCCAAAGGCAGACCGCATTACTGTCACAAAGTCTAGG
GATCTGAGCCACTGCCAGGAGAGAATTGTGAAGGACATTGGTTTGGCATACACTGAAACA
TGTGTGGAGTGCACTAAGGTATTGATCTTATTCTGTAGTGTTTTGAATATAAAAAATAGA
CATTCTCTAGAGCAAAAAAGACAATCACCTTTTTTTTCTCAACAGAGGATCAAAAGTCTG
ATTGAAACTGCAAATTATAACTACATCATAAAACCATCTGACAGTGGTGCACTGATTGCT
GAAGCAACAGTTAAGGAAGTACATCAGTTCTCACCCTTCAATGAGATCCATGGTGCTGCA
ATGATGGAAGCAACGTATGACTGTCTCTCTGTGATTTAGACAGATATATGTAGTATACTC
CCAGCACATTTCATTACTTAAAAAAAAATTAGGTACTAAAGTGTTTTCTTGTTACAGGCA
AAGCTTGGTTTTTGTTGAATTTGAGAAGGCCCCTGTTGTTCCAGTCAAAGCTGATTACAT
GCCCCGTGGATCCCTGCAGTATGAGTTTGCAACTGAGATTCTTCAGACCCCCATTCAACT
CACAAAGATTAGCAATGCACCAGCCCAGGTAAATTAATGCATAAATATATCCGCTCCTGA
TCATATAAATGTATCCACAGTTATAACTTATATAGTTATAACATATATATGACATATTTT
TATTCTTACTAGATAAAGGAGGTCTTGAAACACTTGGTTGAAAACAATGTGGCCATGGTC
CATGATGATGCTCCACTGAAGTTTGTTCAGCTTGTCCAGTTCCTACGTGCCGCCACACTA
AAGGACACTGAAGCCATCTGGGCTCAGTTCAAGGACAAGCCAGTCTACAGGTAAAATGTT
AAGAAATGTTTCTAGGTGCAATATAGTTAAATACTAATGTTTAAGAAAAATCTAAATAAT
TGTTTAATAATTGTTAGGCGCTGGCTTCTGGATACTCTTCCTGCTGTAGCTACACCAGTC
GTTCTAAAGTTCATCAAGGAGAAGTTCCTGGCTGGTGAATTCACTCTTACCGAGTTCATT
CCAACTCTTGTGGTTGCTTTGCAAATGGTCCCTGCTGATTTGGAGACCATCCAGTGGACA
GCTGTGGGTATATCTAACTTTGCTCTAAAGCACTATCCTTAAAATTTAGCATACACTGCA
AAAAAATTATTTGTCTTGTGTCTAGTTTAACACTCTTAAATTAAGAAGCATTTTCTTCAC
AAGTAAAACGAGAGGGGTCTGGCTGCAGACCTGGTGCAGTGCAATCTGTGCTGCATCCAA
TGCTTTTTAATGGGCTCCCCCAACCCTACCCGTCACAGTGATGTCACTAGCTCCATTTGA
GTGCATTGTGTCTGACATTGCATCGCTGACTGATGCAGTCTCAGCTTGCATCATAAAGGC
TGCATCCAGATACTATTGAGTAAAACAGATTTTCTTGTTTTTAGAAATGCCAAAATTAAG
TGTTTTTTAGGACATGCAACAATCTTCCAATAGGGTTAGAAAAAAAACTTTAACTTTTTG
AATTTTAACATTATTTCTAATAACGAGAGAAATTTTAATTGTCAGGAAAATGCTTTCTGA
TTTAAGAATTTAATTGACATTTGGACTGGAAACAAAAAAGTAAGAAAAACTTTTTTTGTG
TGTGTAAGCTAATCTAACGCTAACATCTTTTGAACAGAGTTTGGCTTTGCATGAAAAAAT
TGCCACAATCCCAGCTCTGCGTGAAGTTGTCATGCTTGGATATGGTTCCATGATTGCCAA
GTACTGTGTTGCAGTTCCCACCTGCCCTGCTGAGCTCCTCAGGGTAAGCTCTTCAACAAT
GCCCAATTTCTTGGTAAAAATGTGCTATTTTCTAAAGATGTGTGTATCTTATTCTTCAGC
CCATCCATGAGATCGCCACAGAGGCCATTTCTAAGAATGACATTCCTGAAATCACTTTGG
CTCTGAAAGTTTTGGGCAATGCTGGTCACCCTTCAAGTCTTAAACCTATTATGAAGCTCC
TGCCTGTACTTAAAACTACAGCTTCTGCTCTGCCCATCAGAGTGCAGGTTGATGCCATCT
TGGCCCTGAGGAGCATTGCCAAGAAAGAACCCAAACTGGTAAATTTCAGACATTGTGAAA

ATGAATTGCAGTAAAAATTATTACACACATTTTTATCTTATAATTAACTAACCTTGTTTC
CTGTCTTAGGTTCAGCCTGTTGCCTTGCAGCTTGTTTTGGAAAGAGCTCTGCACCCAGAA
TTGCGCATGGTTGCTTGTATATTGCTGTTTGAGACTAAGCCATCAGTGGCTGTCATGTCC
AGTCTTGCTGGAGCTTTGAGAATGGAGACAAATATGCAGGTTGCAAGCTTTGCCTATTCC
CACATCAAGTCCTTGACCAGAATCACTGCTCCTGATATGTCATCTGTGTAAGTAAAATTG
TTTGTATGGTTTATGTCATATTTTCACACTGGGCTCATTATATACCATTTTTTCTACCAT
AGTGCTGGTGCAGCTAATGTTGCCATCAAGCTTATGAACCGCAAGCTGGACAGACTTAAC
TTCCGTTACAGCAGAGCTTTTCAGTTGGACTTCTATCACAGTGAGTTTAGTGGCTATTTT
CTTTAAGGAGGCTCTCTCAGTGGTAAAGAAGACAAAGTTTTACCCAAAGTGTCTTTCAAA
GTTTTATTTTTTCTAAAGAAGGTCACACAATCATACGTCAAAGACAGTTTCTGCAGAGTG
TGAGACGCCGAAACAGAGTGCGTTCTCTTTTTATCTAGTTTGTAATGCCCACAAGGTCAT
CAGTGACATACAGGATGAATCACAGTTACATCAGCGCTCAAACTTCATTTTTGACCATCT
ATATTTCAGTTCTACCCCCCAATCAAACATATAGTTTGTTAAATCAACTTATTCAGATCT
TGTCTGGTGATAAACACTTATTCTAACTTAAACAGAACATCCATGGAAGAATAAAAGAAC
AGACCTTTATAATGTATATAAATGAGTATATAAAATGTATATAAGATGAATAAAATTAAT
TTTCCACCACAGTGAGTCTACAAAAAAAGACAAAATTAAGAATGCCTTTCATGTAATACC
TATCCATTGATTGTTACCTCTGTCCTTGTGCAGCTCCTCTTATGATTGGAGCTGCTGGTA
GTGCCTACATGATCAATGATGCTGCCACCATCCTGCCCAGAGCTGTCATTGCTAAAGCTC
GTGCTTACTTGGCTGGAGCCGCTACTGATGTTATTGAGGTGAAGCATCAAAAACATCACA
TTTTTGTCTAAAAATTTGATTTATAGTTTACAATATTCTGATAGCCTTATCCTGATGTTT
CCTCTTTAAAAGTTTGGACTGAGAACTGAAGGAATTCAGGAAGCTCTTCTAAAATCTCCT
GCTGCAGATGAAAGTGTTGACCGTATCACTAAAATTAAACGCACCTTAAGAGCTGTAAGT
GATTGGTCTTTTATTATTTGCTCAATAAATGTAAGATTTTTTTAAATAACCATGTTTCTG
TTGCTTACCCCCTTAAGCTCACAAACTGGAAGGCAATGCCAAGCAATAAGCCATTGGCTT
CAGCCTATGTCAAAGTATTTGGGCAAGAAATGGCTTTCGGCAGAATTGACAAGACCATCA
TTGAACAAGCATTACCAGTATGGTGTTCTAGTACATGTATCTACGTTCATGAACATAAAT
CTGAGATCAGTATCACTACACTCAATAACTGAGATTTTACTAAATAAACCTGTATGTATC
ATAAACACTTTCCTCAGATGGTTAATGGACCCAAGCCACGTGCACTGCTAAAGGAGGCTC
TTAAAGCTTTGCAGGAAGGGATTTCCTGGCAGCGTGCCAAACCTCTGCTTGTGGCTGAAG
TGCGCCGTATCTTGCCAACTTCTGTTGGTTTGCCCATGGAGCTCAGTATGTACTCCTCCG
TTGTTGGTGCTGCAACCGTCAATGGTGAGTTTTTTTAAAATTATTATTCCTAAGGTGTA
AAAATGAGTGTTACTTAAGTTTTTTACTTGTATCTTTGTCCCTCTAGTTCAGGCCACCAT
TACACCTCCTCTCCCTGAGCAAATTGAGACCATGACTCTTGAGCAACTGAAAAAGACTGA
TGTTCAATTCAAGGCTGAAGCTAGACCAAGGTAAAGTTTATACCATTATGTATATATACA
CACACAGCGGGGTAAATAAGTCCCCTTGTCTCAGACAACATATTTGGTGCTAACATAAGG
TGCTGTTGACTTGAAATTTTCACTGGATGTCGGTAACAACCAAAGAAATCCATATATGCT
AAGAAAACTAAACTATTTACAAATTAAGTTATGTGTTATAAATTTAATTTACACAAGACA
GAAAATGACAGGCTGAAATCTCCTAGAAGTTATTTAGCAACTCTCTGCTCTTTGTTGTTG
TAAATGAATATTGGCTGCTTCCGTTTAATATCTACATTACCAGGATGTTGAAGATGGATC
CAGGGTGGATATTTCAGCAAGACAATGATCCAAAACACAGCCAAGGAAACTCTCAAAGGG
TTTCAAAGAAAGAAACCTCTAGCTGTAGAATGGCCCAGCCAATCACCTGACTTAAATCCA
ATATAAAATAAAAAAAATCTGATTTGATAAACGAGACCCATAGAACCATCAAGATTTTTA
CATTTTGTTTACATCTGTGAAAAAAAAATCACACCTGATTCAATGCATGTGACTTCATTC
TCCATGGGAGAGGCGTCTTTAAGTTGTCATCACTAAAAAAAAAGAATTTTATATAAGGTA
TTAAATATTCTCACTAGTTCAGTACATTCTCCTTGTAACATTCCATTGTTATGACACATA
ACTAATTTTCAGATTTCTTTTTTGTTTTGTTTTATTTTCATGTTTGTATTTTTGGGGGGG
GGGGTTACTAAAATCTGGTTCAATTCCATGCCAATAGCTCCTAAAATTATTATCCTTAGA
AAATTTTTTTTTTTTTAAAGAATATGTTTTTCCTCCTTTGTGTGTGTGTGTGTGTGTGTG
TGTGTGTGTGTGTGTGTGTGTGTATATATATATATATATATATATATATATATATATATA
TATATATATACACATATATATCATTTTTTTTTTTTTTTTTTTTTTTTTTTTTAATAATA**CC** **22 Fw**
ATCCTCAATCTCGTTCCAGTATTGCTATACATAAATTTGCTGTGATGGGAGTTAACACTG
CCTTCATCCAAGCTGCTGTAATGGCAAGAGGAAAGATCCGTACAATTGCCCCTGGAAAAG
TGGCAGCAAGAGCAGACATTCTCAAGGGCAACTACAAGATTGAGGCTCTGCCTGTTGAAC
TTCCTGAACACATTGCTTCTGCAAGGTAATTAAAACATGGTCATACTAGTTTTGCATATA
AATGAATGAAAATGAAGCAGTTTCTTCAAATATTTGTTGTTCCAACAGCTTTGAGACATT
CGCTTTTGCTAGAGATGTTGAAGATCCCTCTGCTGAGAGAACCGTTCCATTAGTATCTGT **sg22**
GTTGT~~CCCTTCAAAACCGTCAGTCACAAAGACTGGTAAGCCAAACATGCAAATGAATCAA~~
~~ATGCAGCAAGACATTCTTTGATGGTACACAACATATTAATTTCTGTTTCAGTCCTCTGA~~
~~GAATGCTGATTCTGAAATTGAGACGGAAACTCCTGTGAAAGCTCCTGCTCCATTTGACAG~~
~~GACCCTCTGTTATGCTGTCCCATACATTGAAATCAAGGATGTGTTGAGGTGCACTCTCA~~
~~CAATGCTGCTTTCATCAGAAATTCTACTCTGTTCTACATAATTGGACAGCACTCAGCCCCG~~

TGCTGCAGTGGCAAGAGGTATTTTTTAAACAAATTGTCTTTGAATATACATGTTGAATTT 22 Rv
TCAGATTAAATAGTAACTCATTTATATAAAAC**AACTAATTTTGTCATTCTGGCTGT**AGCT
GAAGGTCCTGCAGTTGAAAAACTGGAGTTTGAAGTCCAAGTTGGTCCTAGAGCTGCTGAG
AGGCTTGCTAAGCAAATCAACATCATTGATGATGAAACTCCAGAAGGAAAGGACTTCCTG
TTGAAACTACGGGAAATCTTGGAGACTGAAAGCAAAAATGCAACTGTCTCTTCTGGAAGC
AGCAGCAGCCGCAGCAGCAGTAGCAGCCGCAGCAGCAGCAGCAGTAGCAGCACCAGCAAC
AGTACCAGTAGCAGCAGCTCAAGCTCAAGCTCAAGCTCTTCCATGTCCAGCTCTCGTTGG
TCTAAGGTGAGGACATGGAATCTTGCTTGATATATCATATAACATTTGTATCATAGATTT
TTATTAATTTTATTAATTCTCATTCCTTTCAAATGCTTTTCAGACTCTCACTAAAATGGA
AGCCTTCAGGAAATTCCACAAGGATCAGGTAAATACTCAACACCTTTTACAATGTTTTAA
AGATTTTATTCCCATATATATATATACGTGTGTTTGTGTGTCTAGAGAGAAGAGAAGTAATG
TTAATACTACCCAAACAATTTACTTGCAGTATAAGACACATCATGGAGACTCAAAAAGCA
GTAGAAGCACTGGATCTAGTCTTGAGCAAATCCAGAAACAGGTAAATCAATTTCAGTTTT
TGTTATTTGTCCTAAAAAACTATAAATTTTAAGCCTCATGAATTAACTGCTTATTTCTGA
ACTCATCATTTCAGTCTAGATATCTTGGGAATACTGTTCCACCTGTTTTTGCTATCATCG
CCCGTGCTGTAAGAGTTGACCGGAAGTTGTTGGGATACCAGTTTGTGGCTTTCTTTGACA
AGCCATCTTCAAGAGTGCAGTTCATTGCTTCTTCCATTGCTGAGAATGACAACTTTAAGT
TCTGTGCGGATGGTGTCCTGCTGAGCAAGCACAAAGTCACTGTAAGTTCAAAGATAAGAT
GTCTGAAAATCTTTCAAAAAGCCAAAAATAGCTGACTCTCTCCAAGATGTTCTGCTAGTT
TATAATGTAAATACAGTACCTGTTTCTATTTAGTCCAAGGTTACCTGGGGTGCAGAGTGT
AAAGAATATGCAGTGACTACTAAAGCCGAGGCTGGACTCCTTGGAGAATTCCCAGCTTTC
CGTCTAGAGTGGGAATGGGAAAGGCTTCCAATTATTTTCACCACCTACGCCAAAAAGTAA
ACAATAATGAAGAACTGCTTTGAAGTAACATTACAAGTGTTATGTTTTGAAGACTTTTTA
ACATATTCTTTTTTTCAACAGGCTGTCTAAGCACATTCCTATGGCAGCTTTGCAAGCAGG
ATTTAATGTTGAGAGAGCTAAGAACAGCGAGAAAGAGTTAGAACTTACAGTGGCCTTGCC
ATCTAAGAGAACACTGAATGTAATTGTTAGGGTTCCAGAGGTGAGTGGCCAGCAATTGAC
TCACTAAGATAAACATAAGGGGGAAAATTATTGTAACAATTATATATTTCCTTTAGATGA
CAATGTCAAGGATGGATATTCCTCTCCCAGTTACTTTCCCTATTAATCCAGATGGAACCT
TTGATGTTCATTTTTATGAGGATATTTACTTCAGAGCCCAAAACTATATCTACGATTACA
CCACTGGTAAAATTGGTTGACATTACATGCTTAAATGATCGCATCAAAATAACTTCCCAT
TAAATATATTCTGAAAGCTTATACTGCTTTTAATCCTTTTTAGCTCAATGCAGCATGATG
CAGGATACAATCAGCACCTTCAACAACAAAACATACAAGAATGAAATGCCTATTTCCTGC
TACCAAGTTTTAGCCCAGGATTGCACATCTGAGCTGAAATTTGTTGCTCTGTTGAAGAAG
GACGAAGAGTCTGAAAAGACCCACCTGAATGTTAAACTTGTTGACATGTAAGAACTCTCT
ATATACATTTATTCTGCATTTGTTTTAAATTGCGCTGATCATTTAATTCATACTATATTA
AACTGATCACATGTCTTTAACAGTGATATTGACCTGTATACTTTGGGCACTGATGCAAAA 23 Fw
GTTAAAATTAATGCACTGGAAGTTCCCAT**CAGCAGCCTTCCTTATCAG**CATCCCTCAGGT
AGGTTCAAATGTGCATTTGGTCATTTACTACACATAACATGTTCAGATCATGTAAATTAC
AATGCATTTTTGTTTTAGGCTCCATCCAGATCAGAGAGAAGGCTGATGGTTTGTCACTTT
ATGCTCCTAGTCTTGGGCTTCATGAAGTCTACTTTGCCAATGGTGACTGGAAGGTGAATG
CAAGTTATTGAGTATTTATCAGATACTGATTTCTATTATGCTTGAGAACGTTCTGATACA
AACATTTTATCCAAGATCCAAGTTGCAGACTGGATGAAGGGACAGACTTGTGGACTTTGT sg23
GGAAAGGCTGATGGAGAAATCAAACAGGAGTACACCACACCCAGTGGATACCTGACCGAG
AGCTCAGTCAGCTTTGCACACTCATGGGTGCTGCCTGCTGAGAGTTGCCGTGATGCCAGC
CGTAAGAGTTGTCTGACAAGAATTGTGTATTACACACAATGTTTTAAAGACATCTAAAAA
GGAAAAATAAAAATGTATGTATTTATATCCCTTCTTTCTCAGAATGCCGCATGAAACTTG
AATCTGTCAAGTTTGAGAAACAGGTGATTTTGAACGGACAGGAATCAAAA**TGCTATTCCG** 23 Rv
TTGAGCCTGTGCTGCGCTGTCTGCCAGGCTGTGCACCAGTAAGAACCACACCTGTGACAG
TTGGATACCACTGTATGTCCACTGGTAAGTGATTTTACACTTGTTTATAGAATGACTCTA
GTCCATATTTATTAACAACTTGTGTCAGCACTCTTGCATATTTCCAAATTATCTTGAATG
GCTTGATTAGCTGCTTTAGCTGGGCTTTATTATCGCTGGAGCTAAACTCTACAGGATAGT
GAGTCCCTAGGAGTAGGCTAAACACTTCTACTATAGCATGTTATGCAGATGTCACCAATC
TCAGTTCTGCTCGTGCAGAGTTTAGCTCCAATCTACTTCAACACACCTAATTGGACACTT
TAAGTATACCTAGATTAGTTGGTTCAGGTTTGTTTGATTATTTTTTAAGCTATAATCTAC
AAGAATCTGGCTCTTCAGAACAGAGTGGGGTGACCCCTGGTATAGTGAGTCTCCCGAAAC
ATGTATTAAGACATCTGCTATACAGCACAGTCAGTTTTCAGGACCAAGTTTAAGACCTTT
ACTATACAGGATAATGAGTCTGAAGGAGCAGGCTTGAAGACCTCTGCTATACAGGATAAT
GAGGTCCAGAAGCAGGACTGAAGATCCCTGCTATACTGAACATTGAGTCCCTAGAATCAG
GATTGATTACCTCTGCTATAGAAGATGGTTAGTTCTCTGGAGCTGGATTGGCAATTTTTA
CATATTAATCTAGTCTCCAGAAGCAGGGTTAAAGTCATCTAATATAGAGGATGATAAGTC
TTTATGAACAGATTTGAAGATCCCAGCTTTACAGAATATTGAGTCAGCAGGAGCAGGGTT

GAAGACCTCTGCTATACAGGTTAGTGATTCTCCAGGATCAGGATTGAAGATCTCTGTATA
CAGAATAGTATGTTTCCAGGAGCAGGGTTAAAAACCTCTGCTATATAGAATAGTGAGTTT
CCAGGTGCAGAAAACCTATGGCATATAGTGATTTTCCAGGAGCAGAATAGAAAAACTCTG
CTATACAGAATAGTAAGTCCACAGTTGCAGGTTTGTAGATATCTGCTATACAGGATAGTG
TGTCTCCAGGAGCAGGGTTTAAGACCTCTGCTTTACCCAACTGTGGGTCCCCAGGAGTGG
GAACAAAAAATATTTTAGTCTAGATATTCTAAAATAGCCCTGCTTGAAATGGCACCCTTA
ACAACATTTTGCAAACTAATATGACTCAAGTTCAGTAATTCTCAACTGACCTTGCTTGAG
AAAGCAGTTTTACATTTTACAGTTAAGCACTGGTATATTGCTTTGCACTACATAGCCCAA
CAATATGCAAATGTATTAACTTTGTTAACAAATTTGTTGTTTTGGTTTTTAAATGATGTT
TTAATACCCTGTGTTACAGCCTCTAATCTCAACATGCTTGCTGGAATCTACGAGAAGAGT
GTAGATTTGAGAGAGACAACAGATGCTCACGTGGCCTGTCGCTGCACTGAGCAGTGTGCT
TAAATCCACTTCTTGGCTCAGTAGAGTATAACAACATTCTCTGAATCTGCTGTTACTTGT
TTAGAATAAATCTGAATAAGCAATCTCAAAATCTA

S1 Fig. B) Position of target sites and screening primers on *vtg2* cDNA. Sequence regions corresponding to each yolk protein domain are indicated with the following color highlighted text pointed out with brackets; LvH: plain text PV: orange, LvL: green, Bc: red, Ct: purple. Target sites are indicated in magenta highlighted text along with corresponding labels to the right of the page in front of brackets. sg21, sg22, and sg23: single guide RNAs (sgRNAs) for target sites 1, 2, and 3 for *vtg2*, respectively. Introduced mutation is indicated by strikethrough text.

>*vtg2*(cDNA)

ACAAACCACCAGCCATGAGAGCTGTTGTGCTTGCCTTGACTCTAGCCTTAGTGGCGAGTC
AACAGAACAAACTTGTTCCTGAGTTTGCCCATGATAAGACCTATGTGTACAAGTATGAGG
CTCTACTCTTGAGTGGTATTGTTCAAGAAGGACTGGCCAAAGCAGGTATTCAAATCAAGA
GCAAGGTTCTCCTCAGTGCCGCAACAGAGAATACCTTCCTGTTGAAGTTTGTGGATCCTC
TTTTCTACGAGTATGCTGGCACTTGGCCCAAGGATCAAGTTTTTCCTGCCACTAAGCTCA **sg21**
ACTCAGCACTGGCTGCTCAGCTTCAGACTCCCATCAAGTTTGAGTACGCTAATGGTGTGG
TTGGCAAGGTTTTTGCACCAGCAGGAGTCTCTCCCTCAGTCTTGAACTTGTACAGAGGTA
TCCTCAACATCCTTCAGCTTAACTTTAAGAAGACCCAGAACATCTACGAGCTGCAAGAGG
CTGGAGCTCAGGGAGTTTGCAAAACTCAGTATGTCATCAGTGAGGATCCAAAGGCAGACC
GCATTACTGTCACAAAGTCTAGGGATCTGAGCCACTGCCAGGAGAGAATTGTGAAGGACA
TTGGTTTGGCATACACTGAAACATGTGTGGAGTGCACTAAGAGGATCAAAAGTCTGATTG
AAACTGCAAATTATAACTACATCATAAAACCATCTGACAGTGGTGCACTGATTGCTGAAG
CAACAGTTAAGGAAGTACATCAGTTCTCACCCTTCAATGAGATCCATGGTGCTGCAATGA
TGGAAGCAACGCAAAGCTTGGTTTTTGTTGAATTTGAGAAGGCCCCTGTTGTTCCAGTCA
AAGCTGATTACATGCCCCGTGGATCCCTGCAGTATGAGTTTGCAACTGAGATTCTTCAGA
CCCCCATTCAACTCACAAAGATTAGCAATGCACCAGCCCAGATAAAGGAGGTCTTGAAAC
ACTTGGTTGAAAACAATGTGGCCATGGTCCATGATGATGCTCCACTGAAGTTTGTTCAGC
TTGTCCAGTTCCTACGTGCCGCCACACTAAAGGACACTGAAGCCATCTGGGCTCAGTTCA
AGGACAAGCCAGTCTACAGGCGCTGGCTTCTGGATACTCTTCCTGCTGTAGCTACACCAG
TCGTTCTAAAGTTCATCAAGGAGAAGTTCCTGGCTGGTGAATTCACTCTTACCGAGTTCA
TTCCAACTCTTGTGGTTGCTTTGCAAATGGTCCCTGCTGATTTGGAGACCATCCAGTGGA
CAGCTAGTTTGGCTTTGCATGAAAAAATTGCCACAATCCCAGCTCTGCGTGAAGTTGTCA
TGCTTGGATATGGTTCCATGATTGCCAAGTACTGTGTTGCAGTTCCCACCTGCCCTGCTG
AGCTCCTCAGGCCCATCCATGAGATCGCCACAGAGGCCATTTCTAAGAATGACATTCCTG
AAATCACTTTGGCTCTGAAAGTTTTGGGCAATGCTGGTCACCCTTCAAGTCTTAAACCTA
TTATGAAGCTCCTGCCTGTACTTAAAACTACAGCTTCTGCTCTGCCCATCAGAGTGCAGG **LvH**
TTGATGCCATCTTGGCCCTGAGGAGCATTGCCAAGAAAGAACCCAAACTGGTTCAGCCTG
TTGCCTTGCAGCTTGTTTTGGAAAGAGCTCTGCACCCAGAATTGCGCATGGTTGCTTGTA
TATTGCTGTTTGAGACTAAGCCATCAGTGGCTGTCATGTCCAGTCTTGCTGGAGCTTTGA
GAATGGAGACAAATATGCAGGTTGCAAGCTTTGCCTATTCCCACATCAAGTCCTTGACCA
GAATCACTGCTCCTGATATGTCATCTGTTGCTGGTGCAGCTAATGTTGCCATCAAGCTTA
TGAACCGCAAGCTGGACAGACTTAACTTCCGTTACAGCAGAGCTTTTCAGTTGGACTTCT
ATCACACTCCTCTTATGATTGGAGCTGCTGGTAGTGCCTACATGATCAATGATGCTGCCA
CCATCCTGCCCAGAGCTGTCATTGCTAAAGCTCGTGCTTACTTGGCTGGAGCCGCTACTG
ATGTTATTGAGTTTGGACTGAGAACTGAAGGAATTCAGGAAGCTCTTCTAAAATCTCCTG
CTGCAGATGAAAGTGTTGACCGTATCACTAAAATTAAACGCACCTTAAGAGCTCTCACAA
ACTGGAAGGCAATGCCAAGCAATAAGCCATTGGCTTCAGCCTATGTCAAAGTATTTGGGC
AAGAAATGGCTTTCGGCAGAATTGACAAGACCATCATTGAACAAGCATTACCAATGGTTA
ATGGACCCAAGCCACGTGCACTGCTAAAGGAGGCTCTTAAAGCTTTGCAGGAAGGGATTT
CCTGGCAGCGTGCCAAACCTCTGCTTGTGGCTGAAGTGCGCCGTATCTTGCCAACTTCTG
TTGGTTTGCCCATGGAGCTCAGTATGTACTCCTCCGTTGTTGGTGCTGCAACCGTCAATG
TTCAGGCCACCATTACACCTCCTCTCCCTGAGCAAATTGAGACCATGACTCTTGAGCAAC
TGAAAAAGACTGATGTTCAATTCAAGGCTGAAGCTAGACCAAGTATTGCTATACATAAAT
TTGCTGTGATGGGAGTTAACACTGCCTTCATCCAAGCTGCTGTAATGGCAAGAGGAAAGA
TCCGTACAATTGCCCCTGGAAAAGTGGCAGCAAGAGCAGACATTCTCAAGGGCAACTACA
AGATTGAGGCTCTGCCTGTTGAACTTCCTGAACACATTGCTTCTGCAAGCTTTGAGACAT
TCGCTTTTGCTAGAGATGTTGAAGATCCCTCTGCTGAGAGAACCGTTCCATTAGTATCTG **sg22**
TGTTGT~~CCCTTCAAAACCGTCAGTCACAAAGACTGTCCTCTGAGAATGCTGATTCTGAAA~~
~~TTGAGACGGAAACTCCTGTGAAAGCTCCTGCTCCATTTGACAGGACCCCTCTGTTATGCTG~~
~~TCCCATACATTGAAATCAAGGCATGTGTTGAGGTGCACTCTCACAATGCTGCTTTCATCA~~
~~GAAATTCTACTCTGTTCTACATAATTGGACAGCACTCAGCCCCGTGCTGCAGTGGCAAGAG~~

~~CTGAAGGTCCTGCAGTTGAAAAACTGGAGTTTGAAGTCCAAGTTGGTCCTAGAGCTGCTG~~
~~AGAGGCTTGCTAAGCAAATCAACATCATTGATGATGAAACTCCAGAAGGAAAGGACTTCC~~
~~TGTTGAAACTACGGGAAATCTTGGAGACTGAAAGCAAAAATGCAACTGTCTCTTCTGGAA~~
~~GCAGCAGCAGCCGCAGCAGCAGTAGCAGCCGCAGCAGCAGCAGCAGTAGCAGCACCAGCA~~ Pv
~~ACAGTACCAGTAGCAGCAGCTCAAGCTCAAGCTCAAGCTCTTCCATGTCCAGCTCTCGTT~~
~~GGTCTAAGACTCTCACTAAAATGGAAGCCTTCAGGAAATTCCACAAGGATCAGTATAAGA~~
~~CACATCATGGAGACTCAAAAAGCAGTAGAAGCACTGGATCTAGTCTTGAGCAAATCCAGA~~
~~AACAGTCTAGATATCTTGGAATACTGTTCCACCTGTTTTTGCTATCATCGCCCGTGCTG~~
~~TAAGAGTTGACCGGAAGTTGTTGGATACCAGTTTGTGGCTTTCTTTGACAAGCCATCTT~~
~~CAAGAGTGCAGTTCATTGCTTCTTCCATTGCTGAGAATGACAACTTTAAGTTCTGTGCGG~~
~~ATGGTGTCCTGCTGAGCAAGCACAAAGTCACTTCCAAGGTTACCTGGGGTGCAGAGTGTA~~ LvL
~~AAGAATATGCAGTGACTACTAAAGCCGAGGCTGGACTCCTTGGAGAATTCCCAGCTTTCC~~
~~GTCTAGAGTGGAATGGAAAGGCTTCCAATTATTTTCACCACCTACGCCAAAAAGCTGT~~
~~CTAAGCACATTCCTATGGCAGCTTTGCAAGCAGGATTTAATGTTGAGAGAGCTAAGAACA~~
~~GCGAGAAAGAGTTAGAACTTACAGTGGCCTTGCCATCTAAGAGAACACTGAATGTAATTG~~
~~TTAGGGTTCCAGAGATGACAATGTCAAGGATGGATATTCCTCTCCCAGTTACTTTCCCTA~~
~~TTAATCCAGATGGAACCTTTGATGTTCATTTTTATGAGGATATTTACTTCAGAGCCCAAA~~
~~ACTATATCTACGATTACACCACTGCTCAATGCAGCATGATGCAGGATACAATCAGCACCT~~
~~TCAACAACAAAACATACAAGAATGAAATGCCTATTTCCTGCTACCAAGTTTTAGCCCAGG~~
~~ATTGCACATCTGAGCTGAAATTTGTTGCTCTGTTGAAGAAGGACGAAGAGTCTGAAAAGA~~
~~CCCACCTGAATGTTAAACTTGTTGACATTGATATTGACCTGTATACTTTGGCCACTGATG~~ Ct
~~CAAAAGTTAAAATTAATGGACTGGAAGTTCCCATCAGCAGCCTTCCTTATCAGCATCCCT~~
~~CAGGCTCCATCCAGATCAGAGAGAAGGCTGATGGTTTGTCACTTTATGCTCCTAGTCTTG~~
~~GGCTTCATGAAGTCTACTTTGCCAATGGTGACTGGAAGATCCAAGTTGCAGACTGGATGA~~
~~AGGGACAGACTTGTGGACT~~TTGTGGAAAGGCTGATGGAGAAATCAAACAGGAGTACACCA **sg23**
CACCCAGTGGATACCTGACCGAGAGCTCAGTCAGCTTTGCACACTCATGGGTGCTGCCTG
CTGAGAGTTGCCGTGATGCCAGCCAATGCCGCATGAAACTTGAATCTGTCAAGTTTGAGA
AACAGGTGATTTTGAACGGACAGGAATCAAAATGCTATTCCGTTGAGCCTGTGCTGCGCT Bc
GTCTGCCAGGCTGTGCACCAGTAAGAACCACACCTGTGACAGTTGGATACCACTGTATGT
CCACTGCCTCTAATCTCAACATGCTTGCTGGAATCTACGAGAAGAGTGTAGATTTGAGAG
AGACAACAGATGCTCACGTGGCCTGTCGCTGCACTGAGCAGTGTGCTTAAATCCACTTCT
TGGCTCAGTAGAGTATAACAACATTCTCTGAATCTGCTGTTACTTGTTTAGAATAAATCT
GAATAAGCAATCTCAAAATCTA

S1 Fig. C) Predicted translational frames after deletion on *vtg2*. 5'3'Frame 3 is considered as the potential translation following the introduced mutation.

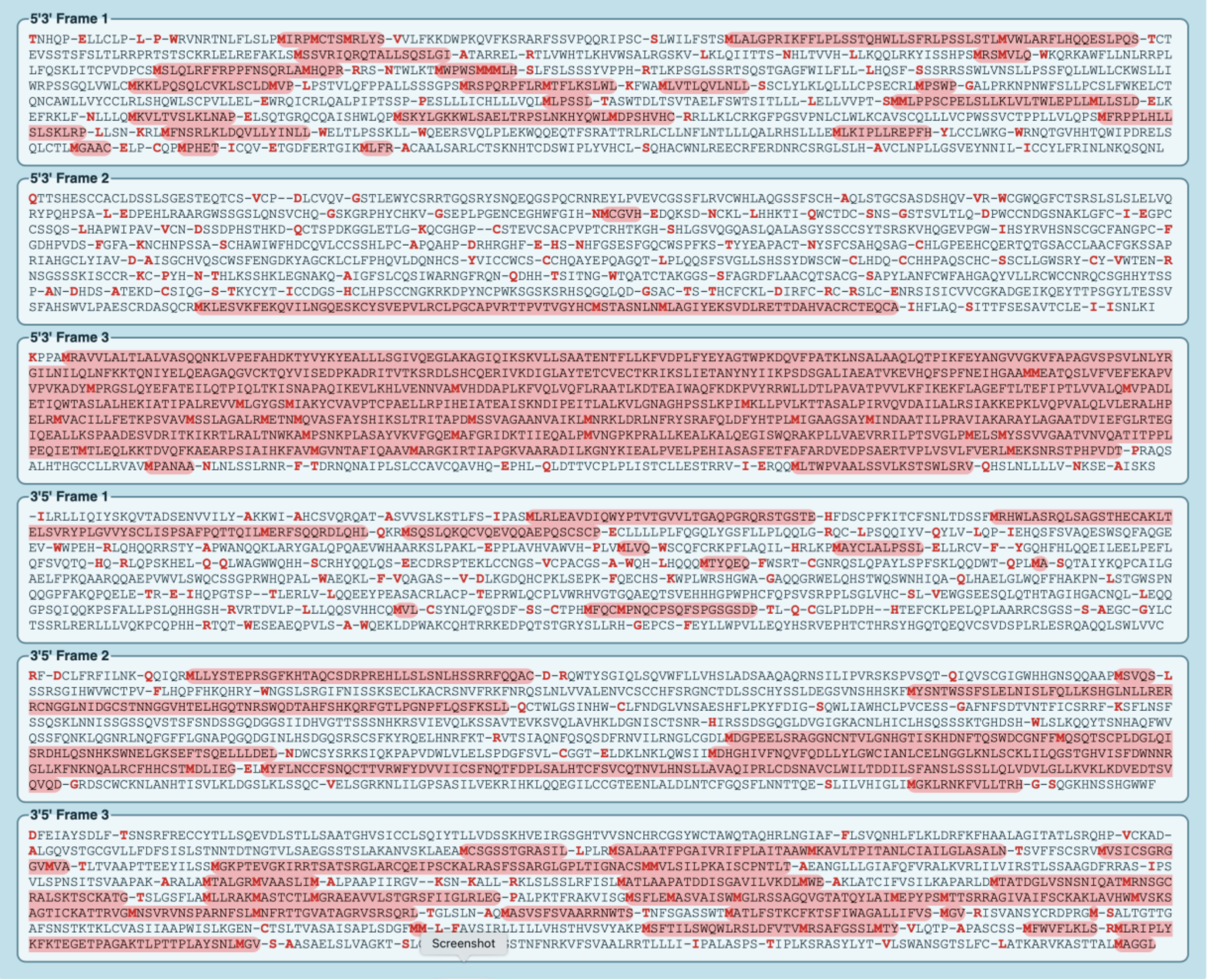

S1 Fig. D) Position of the introduced mutation on Vtg2 peptide sequence. Regions corresponding on each yolk protein domain are indicated in the following color highlighted text; LvH: plain text, Pv: orange, LvL: green, Bc: red, Ct: purple. Introduced mutation is indicated by strikethrough text.

>ENSDART00000061165 peptide: ENSDARP00000061164
KPPAMRAVVLALTLALVASQQNKLVPEFAHDKTYVYKYEALLLSGIVQEGLAKAGIQIKSKVLLSAATE
NTFLLKFVDPLFYEYAGTWPKDQVFPATKLNSALAAQLQTPIKFEYANGVVGKVFAPAGVSPSVLNLYR
GILNILQLNFKKTQNIYELQEAGAQGVCKTQYVISEDPKADRITVTKSRDLSHCQERIVKDIGLAYTET
CVECTKRIKSLIETANYNYIIKPSDSGALIAEATVKEVHQFSPFNEIHGAAMMEATQSLVFVEFEKAPV
VPVKADYMPRGSLQYEFATEILQTPIQLTKISNAPAQIKEVLKHLVENNVAMVHDDAPLKFVQLVQFLR
AATLKDTEAIWAQFKDKPVYRRWLLDTLPAVATPVVLKFIKEKFLAGEFTLTEFIPTLVVALQMVPADL
ETIQWTASLALHEKIATIPALREVVMLGYGSMIAKYCVAVPTCPAELLRPIHEIATEAISKNDIPEITL
ALKVLGNAGHPSSLKPIMKLLPVLKTTASALPIRVQVDAILALRSIAKKEPKLVQPVALQLVLERALHP
ELRMVACILLFETKPSVAVMSSLAGALRMETNMQVASFAYSHIKSLTRITAPDMSSVAGAANVAIKLMN
RKLDRLNFRYSRAFQLDFYHTPLMIGAAGSAYMINDAATILPRAVIAKARAYLAGAATDVIEFGLRTEG
IQEALLKSPAADESVDRITKIKRTLRALTNWKAMPSNKPLASAYVKVFGQEMAFGRIDKTIIEQALPMV
NGPKPRALLKEALKALQEGISWQRAKPLLVAEVRRILPTSVGLPMELSMYSSVVGAATVNVQATITPPL
PEQIETMTLEQLKKTDVQFKAEARPSIAIHKFAVMGVNTAFIQAAVMARGKIRTIAPGKVAARADILKG
NYKIEALPVELPEHIASASFETFAFARDVEDPSAERTVPLVSVL~~SLQNRQSQRLSSENADSEIETETPV~~
~~KAPAPFDRTLCYAVPYIEIKGCVEVHSHNAAFIRNSTLFYIIGQHSARAAVARAEGPAVEKLEFEVQVG~~
~~PRAAERLAKQINIIDDETPEGKDFLLKLREILETESKNATVSSGSSSSRSSSSSRSSSSSSSTSNSTSS~~
~~SSSSSSSSSSMSSSRWSKTLTKMEAFRKFHKDQYKTHHGDSKSSRSTGSSLEQIQKQSRYLGNTVPPVF~~
~~AIIARAVRVDRKLLGYQFVAFFDKPSSRVQFIASSIAENDNFKFCADGVLLSKHKVTSKVTWGAECKEY~~
~~AVTTKAEAGLLGEFPAFRLEWEWERLPIIFTTYAKKLSKHIPMAALQAGFNVERAKNSEKELELTVALP~~
~~SKRTLNVIVRVPEMTMSRMDIPLPVTFPINPDGTFDVHFYEDIYFRAQNYIYDYTTAQCSMMQDTISTF~~
~~NNKTYKNEMPISCYQVLAQDCTSELKFVALLKKDEESEKTHLNVKLVDIDIDLYTLGTDAKVKINGLEV~~
~~PISSLPYQHPSGSIQIREKADGLSLYAPSLGLHEVYFANGDWKIQVADWMKGQTCG~~LCGKADGEIKQEY
TTPSGYLTESSVSFAHSWVLPAESCRDASQCRMKLESVKFEKQVILNGQESKCYSVEPVLRCLPGCAPV
RTTPVTVGYHCMSTASNLNMLAGIYEKSVDLRETTDAHVACRCTEQCA

S1 Table. Proteins differentially regulated in the *vtg2*-mutant eggs. List of the 259 proteins were considered to be differenally regulated between wild type (Wt) and *vtg2*-mutant eggs whose distribution among various functional categories is illustrated in **Fig 8**. These include proteins downregulated in *vtg2-mutant* eggs (Wt: N=83) and those upregulated in *vtg2*-mutant eggs (*vtg2*-mutant: N=176) with N-SC ≥1-fold relative to values for Wt eggs. Only proteins that were detected in at least 4 experimental individuals were considered in this study. For each protein, the Ensembl Protein ID and associated gene, transcript and protein name, functional category, relative abundance (down- or upregulated in *vtg2*-mutant), fold-difference in N-SC between groups (if available), and significance of differences (p < 0.05 and Benjamini Hochberg corrected p < 0.25) is shown. Color shading corresponds to that used to designate functional categories in **Fig 8**.

	Ensembl Protein ID	Associated Gene Name	Associated Transcript Name	Protein full name	Functional Category	Relative Abundance in mutant	Fold Difference	p < 0.05	BH p < 0.25
1	ENSDARP00000006510	pgm1	pgm1-201	Phosphoglucomutase 1	Energy metabolism	upregulated	#DIV/0!	0,02	
2	ENSDARP00000019888	tuba1a	tuba1a-201	Tubulin, alpha 1a	Cell cycle, division, growth and fate	upregulated	#DIV/0!	0,02	
3	ENSDARP00000023177	cfl1	cfl1-201	Cofilin 1	Cell cycle, division, growth and fate	upregulated	#DIV/0!	0,01	
4	ENSDARP00000024752	eef2b	eef2b-001	Eukaryotic translation elongation factor 2b	Protein synthesis	upregulated	#DIV/0!	0,02	
5	ENSDARP00000027118	ywhaba	ywhaba-201	Tyrosine 3-monooxygenase/tryptophan 5-monooxygenase a	Cell cycle, division, growth and fate	upregulated	#DIV/0!	0,01	
6	ENSDARP00000029933	tuba8l4	tuba8l4-201	Tubulin, alpha 8 like 4	Cell cycle, division, growth and fate	upregulated	#DIV/0!	0,03	
7	ENSDARP00000030257	zgc:172218	zgc:172218-201	SUEL-TYPE Lectin	Lectins	upregulated	#DIV/0!	<0.001	
8	ENSDARP00000053303	si:ch211-114n24.6	si:ch211-114n24.6-201	Tubulin alpha chain	Cell cycle, division, growth and fate	upregulated	#DIV/0!	0,02	
9	ENSDARP00000068662	zp3.2	zp3.2-202	Zona pellucida glycoprotein 3, tandem duplicate 2	ZP	upregulated	#DIV/0!	0,00	
10	ENSDARP00000070780	pcna	pcna-201	Proliferating cell nuclear antigen	Cell cycle, division, growth and fate	upregulated	#DIV/0!	<0.001	
11	ENSDARP00000072006	tuba1c	tuba1c-201	Tubulin, alpha 1c	Cell cycle, division, growth and fate	upregulated	#DIV/0!	0,02	
12	ENSDARP00000072581	novel	novel	D-galactoside/L-rhamnose binding SUEL lectin domain	Lectins	upregulated	#DIV/0!	0,00	
13	ENSDARP00000089834	si:ch73-189n23.1	si:ch73-189n23.1-201	SUEL-TYPE Lectin	Lectins	upregulated	#DIV/0!	0,01	
14	ENSDARP00000093490	tuba8l3	tuba8l3-201	Tubulin, alpha 8 like 3	Cell cycle, division, growth and fate	upregulated	#DIV/0!	0,02	
15	ENSDARP00000096076	si:ch211-226h8.6	si:ch211-226h8.6-201	SUEL-TYPE Lectin	Lectins	upregulated	#DIV/0!	0,00	
16	ENSDARP00000096079	si:ch211-226h8.4	si:ch211-226h8.4-201	SUEL-TYPE Lectin	Lectins	upregulated	#DIV/0!	<0.001	
17	ENSDARP00000099845	tuba1b	tuba1b-201	Tubulin, alpha 1b	Cell cycle, division, growth and fate	upregulated	#DIV/0!	<0.001	
18	ENSDARP00000099884	zpax4	zpax4-201	Zona pellucida protein AX 4	ZP	upregulated	#DIV/0!	0,03	
19	ENSDARP00000101100	ywhabb	ywhabb-201	Tyrosine 3-monooxygenase/tryptophan 5-monooxygenase a	Cell cycle, division, growth and fate	upregulated	#DIV/0!	<0.001	
20	ENSDARP00000103071	zgc:173770	zgc:173770-201	Uncharacterized	Other	upregulated	#DIV/0!	0,06	
21	ENSDARP00000103621	si:dkey-240e12.6	si:dkey-240e12.6-201	SUEL-TYPE Lectin	Lectins	upregulated	#DIV/0!	0,01	
22	ENSDARP00000105057	si:dkeyp-46h3.2	si:dkeyp-46h3.2-201	SUEL-TYPE Lectin	Lectins	upregulated	#DIV/0!	0,01	
23	ENSDARP00000105094	tuba1b	tuba1b-201	Tubulin, alpha 1b	Cell cycle, division, growth and fate	upregulated	#DIV/0!	0,02	
24	ENSDARP00000106988	si:dkeyp-98a7.5	si:dkeyp-98a7.5-201	SUEL-TYPE Lectin	Lectins	upregulated	#DIV/0!	0,02	
25	ENSDARP00000107578	si:dkeyp-98a7.4	si:dkeyp-98a7.4-201	SUEL-TYPE Lectin	Lectins	upregulated	#DIV/0!	0,02	
26	ENSDARP00000108507	zgc:152936	zgc:152936-201	SUEL-TYPE Lectin	Lectins	upregulated	#DIV/0!	0,01	
27	ENSDARP00000109226	AL929192.2	AL929192.2-201	D-galactoside/L-rhamnose binding SUEL lectin domain	Lectins	upregulated	#DIV/0!	0,00	
28	ENSDARP00000110519	AL929192.1	AL929192.1-201	D-galactoside/L-rhamnose binding SUEL lectin domain	Lectins	upregulated	#DIV/0!	0,03	
29	ENSDARP00000111434	si:dkeyp-98a7.5	si:dkeyp-98a7.5-201	D-galactoside/L-rhamnose binding SUEL lectin domain	Lectins	upregulated	#DIV/0!	0,02	
30	ENSDARP00000111507	si:ch211-12h2.6	si:ch211-12h2.6-201	SUEL-TYPE Lectin	Lectins	upregulated	#DIV/0!	0,01	
31	ENSDARP00000111784	si:dkeyp-98a7.3	si:dkeyp-98a7.3-201	D-galactoside/L-rhamnose binding SUEL lectin domain	Lectins	upregulated	#DIV/0!	0,02	
32	ENSDARP00000113274	si:ch211-226h8.8	si:ch211-226h8.8-201	SUEL-TYPE Lectin	Lectins	upregulated	#DIV/0!	<0.001	
33	ENSDARP00000114866	si:ch211-226h8.14	si:ch211-226h8.14-201	SUEL-TYPE Lectin	Lectins	upregulated	#DIV/0!	0,00	
34	ENSDARP00000117390	si:ch211-226h8.11	si:ch211-226h8.11-202	SUEL-TYPE Lectin	Lectins	upregulated	#DIV/0!	<0.001	
35	ENSDARP00000120702	si:dkeyp-98a7.4	si:dkeyp-98a7.4-201	SUEL-TYPE Lectin	Lectins	upregulated	#DIV/0!	0,02	
36	ENSDARP00000121868	zgc:172218	zgc:172218-202	SUEL-TYPE Lectin	Lectins	upregulated	#DIV/0!	<0.001	
37	ENSDARP00000122901	ywhaba	ywhaba-202	Tyrosine 3-monooxygenase/tryptophan 5-monooxygenase a	Cell cycle, division, growth and fate	upregulated	#DIV/0!	0,01	
38	ENSDARP00000123950	ywhabb	ywhabb-202	Tyrosine 3-monooxygenase/tryptophan 5-monooxygenase a	Cell cycle, division, growth and fate	upregulated	#DIV/0!	<0.001	
39	ENSDARP00000124587	kpnb1	kpnb1-001	Karyopherin (importin) beta 1	Cell cycle, division, growth and fate	upregulated	#DIV/0!	<0.001	
40	ENSDARP00000125193	si:dkeyp-98a7.7	si:dkeyp-98a7.7-201	SUEL-TYPE Lectin	Lectins	upregulated	#DIV/0!	0,02	
41	ENSDARP00000125214	si:dkeyp-98a7.8	si:dkeyp-98a7.8-201	SUEL-TYPE Lectin	Lectins	upregulated	#DIV/0!	0,02	
42	ENSDARP00000125238	si:dkeyp-98a7.3	si:dkeyp-98a7.3-203	SUEL-TYPE Lectin	Lectins	upregulated	#DIV/0!	0,02	
43	ENSDARP00000125244	si:dkeyp-98a7.3	si:dkeyp-98a7.3-202	SUEL-TYPE Lectin	Lectins	upregulated	#DIV/0!	0,02	
44	ENSDARP00000125496	si:dkeyp-98a7.5	si:dkeyp-98a7.5-202	SUEL-TYPE Lectin	Lectins	upregulated	#DIV/0!	0,02	
45	ENSDARP00000125797	tuba8l4	tuba8l4-203	Tubulin, alpha 8 like 4	Cell cycle, division, growth and fate	upregulated	#DIV/0!	<0.001	
46	ENSDARP00000125988	tuba8l4	tuba8l4-202	Tubulin, alpha 8 like 4	Cell cycle, division, growth and fate	upregulated	#DIV/0!	<0.001	
47	ENSDARP00000109480	vtg5	vtg5-201	Vitellogenin 5	Vitellogenin	upregulated	7,43	0,00	0,11
48	ENSDARP00000024082	atic	atic-201	5-aminoimidazole-4-carboxamide ribonucleotide formyltrans	Cell cycle, division, growth and fate	upregulated	7,00	0,05	0,24
49	ENSDARP00000070667	zp3e	zp3e-201	Zona pellucida glycoprotein 3e	ZP	upregulated	6,17	0,05	0,24
50	ENSDARP00000043687	si:dkey-241l7.2	si:dkey-241l7.2-001	C-type lectin	Lectins	upregulated	4,14	0,04	0,24
51	ENSDARP00000107173	si:dkey-241l7.5	si:dkey-241l7.5-201	C-type lectin	Lectins	upregulated	4,14	0,04	0,24
52	ENSDARP00000109765	si:dkey-241l7.4	si:dkey-241l7.4-201	C-type lectin	Lectins	upregulated	4,14	0,04	0,24
53	ENSDARP00000127204	si:dkey-241l7.2	si:dkey-241l7.2-002	C-type lectin	Lectins	upregulated	4,14	0,04	0,24
54	ENSDARP00000127261	si:dkey-241l7.4	si:dkey-241l7.4-202	C-type lectin	Lectins	upregulated	4,14	0,04	0,24
55	ENSDARP00000127328	si:dkey-241l7.2	si:dkey-241l7.2-003	C-type lectin	Lectins	upregulated	4,14	0,04	0,24
56	ENSDARP00000127453	si:dkey-241l7.5	si:dkey-241l7.5-202	C-type lectin	Lectins	upregulated	4,14	0,04	0,24
57	ENSDARP00000063661	mdh2	mdh2-201	Malate dehydrogenase 2, NAD (mitochondrial)	Energy metabolism	upregulated	4,13	0,02	0,20
58	ENSDARP00000104702	qdprb2	qdprb2-203	Quinoid dihydropteridine reductase b2	Redox/Detox	upregulated	4,06	0,01	0,17
59	ENSDARP00000103251	qdprb2	qdprb2-201	Quinoid dihydropteridine reductase b2	Redox/Detox	upregulated	4,06	0,01	0,17
60	ENSDARP00000049792	gstm.1	gstm.1-201	Glutathione S-transferase mu, tandem duplicate 1	Redox/Detox	upregulated	3,94	0,02	0,19
61	ENSDARP00000107948	CU467646.1	CU467646.1-202	Uncharacterized	Other	upregulated	3,90	0,01	0,17
62	ENSDARP00000008576	si:dkey-46g23.5	si:dkey-46g23.5-201	Alpha 2 macroglobulin	Immune system	upregulated	3,74	0,12	
63	ENSDARP00000073057	hspd1	hspd1-201	Heat shock 60 protein 1	Protein synthesis	upregulated	3,69	0,07	
64	ENSDARP00000107955	hspd1	hspd1-202	Heat shock 60 protein 1	Protein synthesis	upregulated	3,69	0,07	
65	ENSDARP00000098065	nme2b.2	nme2b.2-203	NME/NM23 nucleoside diphosphate kinase 2b, tandem dupl	Cell cycle, division, growth and fate	upregulated	3,51	0,08	
66	ENSDARP00000101082	nme2b.2	nme2b.2-202	NME/NM23 nucleoside diphosphate kinase 2b, tandem dupl	Cell cycle, division, growth and fate	upregulated	3,51	0,08	
67	ENSDARP00000093898	p4hb	p4hb-001	Procollagen-proline, 2-oxoglutarate 4-dioxygenase (proline	Protein synthesis	upregulated	3,50	0,02	0,20
68	ENSDARP00000073345	si:dkey-46g23.5	si:dkey-46g23.5-202	Alpha 2 macroglobulin	Immune system	upregulated	3,06	0,13	
69	ENSDARP00000066852	f13a1a.1	f13a1a.1-201	Coagulation factor XIII, A1 polypeptide a, tandem duplicate	Immune system	upregulated	2,98	0,24	
70	ENSDARP00000120334	f13a1a.1	f13a1a.1-001	Coagulation factor XIII, A1 polypeptide a, tandem duplicate	Immune system	upregulated	2,98	0,24	
71	ENSDARP00000072687	vtg5	vtg5-001	Vitellogenin 5	Vitellogenin	upregulated	2,87	0,03	0,24
72	ENSDARP00000099319	nme2b.2	nme2b.2-204	NME/NM23 nucleoside diphosphate kinase 2b, tandem dupl	Cell cycle, division, growth and fate	upregulated	2,87	0,07	
73	ENSDARP00000117259	MFAP4 (12 of 14)	MFAP4 (12 of14)-201	Microfibrillar-associated protein 4	Cell cycle, division, growth and fate	upregulated	2,84	0,01	0,17
74	ENSDARP00000100463	zgc:171426	zgc:171426-201	Alpha 2 macroglobulin	Immune system	upregulated	2,69	0,21	
75	ENSDARP00000057284	nasp	nasp-202	Nuclear autoantigenic sperm protein (histone-binding)	Cell cycle, division, growth and fate	upregulated	2,59	0,07	
76	ENSDARP00000112476	qdprb2	qdprb2-201	Quinoid dihydropteridine reductase b2	Redox/Detox	upregulated	2,58	0,09	
77	ENSDARP00000107611	qdprb2	qdprb2-202	Quinoid dihydropteridine reductase b2	Redox/Detox	upregulated	2,58	0,09	
78	ENSDARP00000002544	zp2.5	zp2.5-201	Zona pellucida glycoprotein 2, tandem duplicate 5	ZP	upregulated	2,39	0,05	0,24
79	ENSDARP00000047100	zp2.2	zp2.2-001	Zona pellucida glycoprotein 2, tandem duplicate 2	ZP	upregulated	2,39	0,05	0,24
80	ENSDARP00000090133	zp2.1	zp2.1-201	Zona pellucida glycoprotein 2, tandem duplicate 1	ZP	upregulated	2,39	0,05	0,24
81	ENSDARP00000109385	zp2.6	zp2.6-201	Zona pellucida glycoprotein 2, tandem duplicate 6	ZP	upregulated	2,39	0,05	0,24
82	ENSDARP00000056088	si:zfos-1505d6.3	si:zfos-1505d6.3-201	Zona pellucida glycoprotein 2, tandem duplicate 6 precurso	ZP	upregulated	2,26	0,05	0,24
83	ENSDARP00000025702	tfa	tfa-201	Transferrin-a	Cell cycle, division, growth and fate	upregulated	2,25	0,51	
84	ENSDARP00000100241	tfa	tfa-202	Transferrin-a	Cell cycle, division, growth and fate	upregulated	2,25	0,51	
85	ENSDARP00000117697	nasp	nasp-205	Nuclear autoantigenic sperm protein (histone-binding)	Cell cycle, division, growth and fate	upregulated	2,18	0,10	
86	ENSDARP00000005260	MFAP4 (8 of 14)	MFAP4 (8 of 14)-201	Microfibrillar-associated protein 4	Cell cycle, division, growth and fate	upregulated	2,13	0,07	
87	ENSDARP00000116487	zgc:165539	zgc:165539-204	Uncharacterized	Other	upregulated	2,12	0,35	
88	ENSDARP00000068574	zp2.3	zp2.3-201	Zona pellucida glycoprotein 2, tandem duplicate 3	ZP	upregulated	2,05	0,05	0,24
89	ENSDARP00000059885	ldha	ldha-201	Lactate dehydrogenase A4	Energy metabolism	upregulated	1,99	0,20	
90	ENSDARP00000013329	ldhba	ldhba-201	Lactate dehydrogenase Ba	Energy metabolism	upregulated	1,98	0,09	
91	ENSDARP00000003641	ppiaa	ppiaa-201	Peptidylprolyl isomerase Aa (cyclophilin A)	Protein synthesis	upregulated	1,95	0,10	
92	ENSDARP00000106349	ppiaa	ppiaa-202	Peptidylprolyl isomerase Aa (cyclophilin A)	Protein synthesis	upregulated	1,95	0,10	
93	ENSDARP00000037871	ckma	ckma-201	Creatine kinase, muscle a	Energy metabolism	upregulated	1,94	0,25	
94	ENSDARP00000117584	ckba	ckba-203	Creatine kinase, brain a	Energy metabolism	upregulated	1,94	0,26	
95	ENSDARP00000122552	ckba	ckba-204	Creatine kinase, brain a	Energy metabolism	upregulated	1,93	0,26	
96	ENSDARP00000059365	ckmb	ckmb-201	Creatine kinase, muscle b	Energy metabolism	upregulated	1,93	0,26	
97	ENSDARP00000118106	ldhbb	ldhbb-203	Lactate dehydrogenase Bb	Energy metabolism	upregulated	1,83	0,25	
98	ENSDARP00000095636	ldhbb	ldhbb-201	Lactate dehydrogenase Bb	Energy metabolism	upregulated	1,83	0,25	
99	ENSDARP00000106067	ldhbb	ldhbb-201	Lactate dehydrogenase Bb	Energy metabolism	upregulated	1,83	0,25	
100	ENSDARP00000066429	actc1b	actc1b-201	Actin alpha cardiac muscle 1b	Cell cycle, division, growth and fate	upregulated	1,76	0,34	
101	ENSDARP00000052837	acta1a	acta1a-201	Actin alpha 1, skeletal muscle a	Cell cycle, division, growth and fate	upregulated	1,75	0,32	
102	ENSDARP00000055135	actc1b	actc1b-201	Actin, alpha, cardiac muscle 1b	Cell cycle, division, growth and fate	upregulated	1,75	0,32	
103	ENSDARP00000058628	acta1b	acta1b-201	Actin alpha 1, skeletal muscle b	Cell cycle, division, growth and fate	upregulated	1,75	0,32	
104	ENSDARP00000062369	actc1a	actc1a-201	Actin alpha cardiac muscle 1a	Cell cycle, division, growth and fate	upregulated	1,75	0,32	
105	ENSDARP00000075110	ACTC1	ACTC1-201	Novel actin protein	Cell cycle, division, growth and fate	upregulated	1,75	0,32	
106	ENSDARP00000100434	actc1a	actc1a-203	Actin alpha cardiac muscle 1a	Cell cycle, division, growth and fate	upregulated	1,75	0,32	
107	ENSDARP00000060457	si:dkey-241l7.3	si:dkey-241l7.3-201	C-type lectin	Lectins	upregulated	1,75	0,55	
108	ENSDARP00000127264	si:dkey-241l7.3	si:dkey-241l7.3-202	C-type lectin	Lectins	upregulated	1,75	0,55	
109	ENSDARP00000127402	si:dkey-241l7.3	si:dkey-241l7.3-203	C-type lectin	Lectins	upregulated	1,75	0,55	
110	ENSDARP00000129036	si:dkey-241l7.6	si:dkey-241l7.6-204	C-type lectin	Lectins	upregulated	1,75	0,55	
111	ENSDARP00000060465	si:dkey-241l7.6	si:dkey-241l7.6-201	C-type lectin	Lectins	upregulated	1,75	0,55	
112	ENSDARP00000127254	si:dkey-241l7.6	si:dkey-241l7.6-202	C-type lectin	Lectins	upregulated	1,75	0,55	
113	ENSDARP00000128683	actc1c	actc1c-202	Actin alpha cardiac muscle 1c	Cell cycle, division, growth and fate	upregulated	1,70	0,36	
114	ENSDARP00000124371	acta2	acta2-202	Actin alpha 2, smooth muscle	Cell cycle, division, growth and fate	upregulated	1,67	0,36	
115	ENSDARP00000100195	actc1c	actc1c-201	Actin alpha cardiac muscle 1c	Cell cycle, division, growth and fate	upregulated	1,65	0,37	
116	ENSDARP00000018458	pdia4	pdia4-201	Protein disulfide isomerase family A, member 4	Protein synthesis	upregulated	1,59	0,63	
117	ENSDARP00000072983	eef1g	eef1g-201	Eukaryotic translation elongation factor 1 gamma	Protein synthesis	upregulated	1,58	0,41	
118	ENSDARP00000013441	hsp90b1	hsp90b1-201	Heat shock protein 90, beta (grp94), member 1	Protein synthesis	upregulated	1,51	0,47	
119	ENSDARP00000069198	nots	nots-201	Nothepsin	Protein degradation and synthesis inhibition	upregulated	1,51	0,37	
120	ENSDARP00000017569	tktb	tktb-201	Transketolase b	Energy metabolism	upregulated	1,49	0,40	
121	ENSDARP00000015293	cp	cp-201	Ceruloplasmin	Cell cycle, division, growth and fate	upregulated	1,45	0,12	
122	ENSDARP00000005298	chia.4	chia.4-201	Chitinase, acidic.4	Other	upregulated	1,41	0,64	
123	ENSDARP00000116010	vtg7	vtg7-203	Vitellogenin 7	Vitellogenin	upregulated	1,39	0,31	
124	ENSDARP00000104065	zgc:173837	zgc:173837-201	Uncharacterized	Other	upregulated	1,34	0,76	
125	ENSDARP00000120276	ldhbb	ldhbb-202	Lactate dehydrogenase Bb	Energy metabolism	upregulated	1,27	0,68	
126	ENSDARP00000002844	ahcy	ahcy-201	Adenosylhomocysteinase	Protein degradation and synthesis inhibition	upregulated	1,24	0,80	
127	ENSDARP00000126800	ckbb	ckbb-207	Creatine kinase, brain b	Energy metabolism	upregulated	1,23	0,33	
128	ENSDARP00000129378	zgc:163040	zgc:163040-202	Histone H4 domain	Cell cycle, division, growth and fate	upregulated	1,21	0,65	
129	ENSDARP00000077832	H4C2	H4C2-201	Histone H4	Cell cycle, division, growth and fate	upregulated	1,21	0,65	
130	ENSDARP00000082327	zgc:153409	zgc:153409-201	Histone H4 domain	Cell cycle, division, growth and fate	upregulated	1,21	0,65	
131	ENSDARP00000101311	HIST1H4K	HIST1H4K-001	Histone H4	Cell cycle, division, growth and fate	upregulated	1,21	0,65	
132	ENSDARP00000101467	HIST1H4C	HIST1H4C-001	Histone H4	Cell cycle, division, growth and fate	upregulated	1,21	0,65	
133	ENSDARP00000101862	si:ch211-113a14.10	si:ch211-113a14.10-201	Histone H4 domain	Cell cycle, division, growth and fate	upregulated	1,21	0,65	
134	ENSDARP00000104379	zgc:165555	zgc:165555-201	Histone H4	Cell cycle, division, growth and fate	upregulated	1,21	0,65	
135	ENSDARP00000105059	HIST1H4D	HIST1H4D-201	Histone H4	Cell cycle, division, growth and fate	upregulated	1,21	0,65	
136	ENSDARP00000105168	BX323062	novel	Histone H4 domain	Cell cycle, division, growth and fate	upregulated	1,21	0,65	
137	ENSDARP00000105496	BX323062	novel	Histone H4 domain	Cell cycle, division, growth and fate	upregulated	1,21	0,65	
138	ENSDARP00000106245	hist1h4l	hist1h4l-211	Histone 1, H4, like	Cell cycle, division, growth and fate	upregulated	1,21	0,65	
139	ENSDARP00000106443	HIST1H4I (1 of 2)	HIST1H4I (1 of 2)-201	Histone H4	Cell cycle, division, growth and fate	upregulated	1,21	0,65	
140	ENSDARP00000106496	HIST2H4A	HIST2H4A-001	Histone H4	Cell cycle, division, growth and fate	upregulated	1,21	0,65	
141	ENSDARP00000106634	BX323062	novel	Histone H4 domain	Cell cycle, division, growth and fate	upregulated	1,21	0,65	
142	ENSDARP00000107028	si:dkey-108k21.18	si:dkey-108k21.18-001	Histone H4 domain	Cell cycle, division, growth and fate	upregulated	1,21	0,65	
143	ENSDARP00000107148	BX323062	novel	Histone H4 domain	Cell cycle, division, growth and fate	upregulated	1,21	0,65	
144	ENSDARP00000107547	BX323062	novel	Histone H4 domain	Cell cycle, division, growth and fate	upregulated	1,21	0,65	
145	ENSDARP00000107585	novel	novel	Histone H4 domain	Cell cycle, division, growth and fate	upregulated	1,21	0,65	
146	ENSDARP00000107946	BX323062	novel	Histone H4 domain	Cell cycle, division, growth and fate	upregulated	1,21	0,65	
147	ENSDARP00000107998	novel	novel	Histone H4 domain	Cell cycle, division, growth and fate	upregulated	1,21	0,65	
148	ENSDARP00000108502	CABZ01041214	novel	Histone H4 domain	Cell cycle, division, growth and fate	upregulated	1,21	0,65	
149	ENSDARP00000109044	BX323062	novel	Histone H4 domain	Cell cycle, division, growth and fate	upregulated	1,21	0,65	
150	ENSDARP00000109071	BX323062	novel	Histone H4 domain	Cell cycle, division, growth and fate	upregulated	1,21	0,65	
151	ENSDARP00000109257	HIST1H4I (2 of 2)	HIST1H4I (2 of 2)-001	Histone H4	Cell cycle, division, growth and fate	upregulated	1,21	0,65	
152	ENSDARP00000109299	zgc:163040	zgc:163040-205	Histone H4 domain	Cell cycle, division, growth and fate	upregulated	1,21	0,65	
153	ENSDARP00000109355	BX323062	novel	Histone H4 domain	Cell cycle, division, growth and fate	upregulated	1,21	0,65	
154	ENSDARP00000110935	zgc:163040	zgc:163040-216	Histone H4 domain	Cell cycle, division, growth and fate	upregulated	1,21	0,65	
155	ENSDARP00000111175	HIST1H4H	HIST1H4H-001	Histone H4	Cell cycle, division, growth and fate	upregulated	1,21	0,65	
156	ENSDARP00000111526	HIST1H4F	HIST1H4F-201	Histone H4	Cell cycle, division, growth and fate	upregulated	1,21	0,65	
157	ENSDARP00000111786	zgc:163040	zgc:163040-206	Histone H4 domain	Cell cycle, division, growth and fate	upregulated	1,21	0,65	
158	ENSDARP00000111930	zgc:163040	zgc:163040-214	Histone H4 domain	Cell cycle, division, growth and fate	upregulated	1,21	0,65	
159	ENSDARP00000112034	zgc:163040	zgc:163040-217	Histone H4 domain	Cell cycle, division, growth and fate	upregulated	1,21	0,65	
160	ENSDARP00000129044	zgc:165555	zgc:165555-201	Histone H4 domain	Cell cycle, division, growth and fate	upregulated	1,21	0,65	
161	ENSDARP00000060309	atp5f1b	atp5f1b-201	ATP synthase F1 subunit beta	Energy metabolism	upregulated	1,18	0,77	
162	ENSDARP00000034536	uchl1	uchl1-201	Ubiquitin carboxyl-terminal esterase L1 (ubiquitin thiolester	Protein degradation and synthesis inhibition	upregulated	1,16	0,48	
163	ENSDARP00000015917	ckba	ckba-201	Creatine kinase, brain a	Energy metabolism	upregulated	1,16	0,64	
164	ENSDARP00000092517	ckba	ckba-202	Creatine kinase, brain a	Energy metabolism	upregulated	1,16	0,64	
165	ENSDARP00000072693	vtg6	vtg6-201	Vitellogenin 6	Vitellogenin	upregulated	1,14	0,36	
166	ENSDARP00000089879	ubc	ubc-001	Ubiquitin c	Protein degradation and synthesis inhibition	upregulated	1,13	0,84	
167	ENSDARP00000107110	rps27a	rps27a-201	Ribosomal protein S27a	Protein synthesis	upregulated	1,12	0,85	
168	ENSDARP00000128775	rps27a	rps27a-204	Ribosomal protein S27a	Protein synthesis	upregulated	1,12	0,85	
169	ENSDARP00000060744	uba52	uba52-201	Ubiquitin A-52 residue ribosomal protein fusion product 1	Protein degradation and synthesis inhibition	upregulated	1,12	0,85	
170	ENSDARP00000030819	UBB	UBB-201	Ubiquitin like	Protein degradation and synthesis inhibition	upregulated	1,12	0,85	
171	ENSDARP00000088354	ubb	ubb-201	Ubiquitin B	Protein degradation and synthesis inhibition	upregulated	1,12	0,85	
172	ENSDARP00000106956	UBC	UBC-201	Ubiquitin c	Protein degradation and synthesis inhibition	upregulated	1,12	0,85	
173	ENSDARP00000115483	zgc:171717	zgc:171717-201	SUEL-TYPE Lectin	Lectins	upregulated	1,10	0,90	
174	ENSDARP00000116723	zgc:171717	zgc:171717-202	SUEL-TYPE Lectin	Lectins	upregulated	1,10	0,90	
175	ENSDARP00000120742	eno3	eno3-205	Enolase 3, (beta, muscle)	Energy metabolism	upregulated	1,03	0,93	
176	ENSDARP00000107746	chia.4	chia.4-202	Chitinase, acidic.4	Other	upregulated	1,02	0,98	
177	ENSDARP00000072738	vtg1	vtg1-201	Vitellogenin 1	Vitellogenin	downregulated	0,97	0,57	
178	ENSDARP00000115830	retsatl	retsatl-203	Retinol saturase (all-trans-retinol 13,14-reductase) like	Lipid metabolism	downregulated	0,97	0,74	
179	ENSDARP00000043898	retsatl	retsatl-201	Retinol saturase (all-trans-retinol 13,14-reductase) like	Lipid metabolism	downregulated	0,97	0,72	
180	ENSDARP00000119867	hspa8	hspa8-202	Heat shock protein 8	Protein synthesis	downregulated	0,96	0,91	
181	ENSDARP00000128406	vtg3	vtg3-001	Vitellogenin 3, phosvitinless	Vitellogenin	downregulated	0,96	0,76	
182	ENSDARP00000033907	tpi1a	tpi1a-201	Triosephosphate isomerase 1a	Energy metabolism	downregulated	0,91	0,86	
183	ENSDARP00000115703	eno3	eno3-201	Enolase 3, (beta, muscle)	Energy metabolism	downregulated	0,89	0,78	

184	ENSDARP00000024900	ckbb	ckbb-201	Creatine kinase, brain b	Energy metabolism	downregulated	0,85	0,31	
185	ENSDARP00000112639	actb1	actb1-202	Actin, beta 1	Cell cycle, division, growth and fate	downregulated	0,84	0,54	
186	ENSDARP00000124779	cat	cat-001	Catalase	Redox/detox	downregulated	0,83	0,50	
187	ENSDARP00000017456	hspa5	hspa5-201	Heat shock protein 5	Protein synthesis	downregulated	0,83	0,35	
188	ENSDARP00000115259	actb1	actb1-204	Actin, beta 1	Cell cycle, division, growth and fate	downregulated	0,78	0,39	
189	ENSDARP00000054986	actb1	actb1-201	Actin, beta 1	Cell cycle, division, growth and fate	downregulated	0,78	0,20	
190	ENSDARP00000055193	actb2	actb2-201	Actin, beta 2	Cell cycle, division, growth and fate	downregulated	0,78	0,20	
191	ENSDARP00000122263	actb2	actb2-206	Actin, beta 2	Cell cycle, division, growth and fate	downregulated	0,78	0,20	
192	ENSDARP00000011595	gyg1a	gyg1a-201	Glycogenin 1a	Energy metabolism	downregulated	0,78	0,20	
193	ENSDARP00000023158	vtg3	vtg3-201	Vitellogenin 3, phosvitinless	Vitellogenin	downregulated	0,77	0,08	
194	ENSDARP00000072678	vtg7	vtg7-201	Vitellogenin 7	Vitellogenin	downregulated	0,76	0,20	
195	ENSDARP00000114558	vtg7	vtg7-204	Vitellogenin 7	Vitellogenin	downregulated	0,76	0,20	
196	ENSDARP00000039324	hsc70	hsc70-201	Heat shock cognate 70	Protein synthesis	downregulated	0,74	0,47	
197	ENSDARP00000097305	gyg1b	gyg1b-201	Glycogenin 1b	Energy metabolism	downregulated	0,74	0,49	
198	ENSDARP00000026546	pdia3	pdia3-001	Protein disulfide isomerase family A, member 3	Protein synthesis	downregulated	0,74	0,55	
199	ENSDARP00000129394	pdia3	pdia3-201	Protein disulfide isomerase family A, member 3	Protein synthesis	downregulated	0,74	0,55	
200	ENSDARP00000115012	vtg4	vtg4-202	Vitellogenin 4	Vitellogenin	downregulated	0,72	<0.001	0,01
201	ENSDARP00000120388	actb2	actb2-207	Actin, beta 2	Cell cycle, division, growth and fate	downregulated	0,71	0,28	
202	ENSDARP00000116418	actb1	actb1-205	Actin, beta 1	Cell cycle, division, growth and fate	downregulated	0,71	0,28	
203	ENSDARP00000095458	pfn2	pfn2-201	Profilin 2	Cell cycle, division, growth and fate	downregulated	0,71	0,45	
204	ENSDARP00000118517	gapdh	gapdh-203	Glyceraldehyde-3-phosphate dehydrogenase	Energy metabolism	downregulated	0,71	0,45	
205	ENSDARP00000118100	gapdh	gapdh-204	Glyceraldehyde-3-phosphate dehydrogenase	Energy metabolism	downregulated	0,71	0,45	
206	ENSDARP00000045696	TXN	TXN-201	Thioredoxin	Redox/detox	downregulated	0,69	0,42	
207	ENSDARP00000040396	prdx2	prdx2-201	Peroxiredoxin 2	Redox/detox	downregulated	0,68	0,26	
208	ENSDARP00000013402	cat	cat-201	Catalase	Redox/detox	downregulated	0,67	0,15	
209	ENSDARP00000107673	cat	cat-202	Catalase	Redox/detox	downregulated	0,67	0,15	
210	ENSDARP00000101309	vtg4	vtg4-201	Vitellogenin 4	Vitellogenin	downregulated	0,66	<0.001	0,01
211	ENSDARP00000063799	gapdh	gapdh-201	Glyceraldehyde-3-phosphate dehydrogenase	Energy metabolism	downregulated	0,66	0,25	
212	ENSDARP00000010413	hsp70.3	hsp70.3-201	Heat shock cognate 70-kd protein, tandem duplicate 3	Protein synthesis	downregulated	0,66	0,27	
213	ENSDARP00000030050	hsp70l	hsp70l-201	Heat shock cognate 70-kd protein, like	Protein synthesis	downregulated	0,66	0,27	
214	ENSDARP00000045766	mcm5	mcm5-201	MCM5 minichromosome maintenance deficient 5 (S. cerevis	Cell cycle, division, growth and fate	downregulated	0,66	0,27	
215	ENSDARP00000103854	hsp70.2	hsp70.2-202	Heat shock cognate 70-kd protein, tandem duplicate 2	Protein synthesis	downregulated	0,66	0,27	
216	ENSDARP00000109199	hsp70.1	hsp70.1-202	Heat shock cognate 70-kd protein, tandem duplicate 1	Protein synthesis	downregulated	0,66	0,27	
217	ENSDARP00000004394	si:ch211-199o1.2	si:ch211-199o1.2-201	Heat shock 70 kDa protein-like	Protein synthesis	downregulated	0,66	0,27	
218	ENSDARP00000114162	hspa1b	hspa1b-201	Heat shock protein family A (Hsp70) member 1B	Protein synthesis	downregulated	0,65	0,26	
219	ENSDARP00000090766	hspa8	hspa8-201	Heat shock protein 8	Protein synthesis	downregulated	0,65	0,18	
220	ENSDARP00000073132	hsp70.2	hsp70.2-201	Heat shock cognate 70-kd protein, tandem duplicate 1	Protein synthesis	downregulated	0,65	0,26	
221	ENSDARP00000102412	mcm5	mcm5-202	MCM5 minichromosome maintenance deficient 5 (S. cerevis	Cell cycle, division, growth and fate	downregulated	0,65	0,26	
222	ENSDARP00000094194	vtg3	vtg3-202	Vitellogenin 3, phosvitinless	Vitellogenin	downregulated	0,63	0,02	0,20
223	ENSDARP00000050237	vtg1	vtg1-201	Vitellogenin 1	Vitellogenin	downregulated	0,60	0,02	0,20
224	ENSDARP00000043742	hspa8b	hspa8b-201	Heat shock protein family A (Hsp70) member 8b	Protein synthesis	downregulated	0,59	0,33	
225	ENSDARP00000115226	ckbb	ckbb-204	Creatine kinase, brain b	Energy metabolism	downregulated	0,59	0,14	
226	ENSDARP00000116170	zgc:113984	zgc:113984-201	SUEL-TYPE Lectin	Lectins	downregulated	0,55	0,36	
227	ENSDARP00000121130	zgc:113984	zgc:113984-202	SUEL-TYPE Lectin	Lectins	downregulated	0,55	0,36	
228	ENSDARP00000122705	zgc:113984	zgc:113984-203	SUEL-TYPE Lectin	Lectins	downregulated	0,55	0,36	
229	ENSDARP00000113988	vtg6	vtg6-204	Vitellogenin 6	Vitellogenin	downregulated	0,54	0,01	0,17
230	ENSDARP00000126677	ckbb	ckbb-205	Creatine kinase, brain b	Energy metabolism	downregulated	0,52	0,32	
231	ENSDARP00000003738	eno1a	eno1a-201	Enolase 1a, (alpha)	Energy metabolism	downregulated	0,52	0,50	
232	ENSDARP00000118406	cnp	cnp-005	2,,3,-cyclic nucleotide 3, phosphodiesterase	Cell cycle, division, growth and fate	downregulated	0,48	0,06	0,24
233	ENSDARP00000098561	cnp	cnp-202	2,,3,-cyclic nucleotide 3, phosphodiesterase	Cell cycle, division, growth and fate	downregulated	0,48	0,06	0,24
234	ENSDARP00000117370	cnp	cnp-001	2,,3,-cyclic nucleotide 3, phosphodiesterase	Cell cycle, division, growth and fate	downregulated	0,48	0,06	0,24
235	ENSDARP00000119679	cnp	cnp-010	2,,3,-cyclic nucleotide 3, phosphodiesterase	Cell cycle, division, growth and fate	downregulated	0,48	0,06	0,24
236	ENSDARP00000120541	cnp	cnp-003	2,,3,-cyclic nucleotide 3, phosphodiesterase	Cell cycle, division, growth and fate	downregulated	0,48	0,06	0,24
237	ENSDARP00000114417	cnp	cnp-004	2,,3,-cyclic nucleotide 3, phosphodiesterase	Cell cycle, division, growth and fate	downregulated	0,48	0,06	0,24
238	ENSDARP00000117465	cnp	cnp-009	2,,3,-cyclic nucleotide 3, phosphodiesterase	Cell cycle, division, growth and fate	downregulated	0,48	0,06	0,24
239	ENSDARP00000056380	crp2	crp2-201	C-reactive protein 2	Immune system	downregulated	0,47	0,02	0,22
240	ENSDARP00000095074	cnp	cnp-201	2,,3,-cyclic nucleotide 3, phosphodiesterase	Cell cycle, division, growth and fate	downregulated	0,44	0,03	0,24
241	ENSDARP00000124216	cnp	cnp-007	2,,3,-cyclic nucleotide 3, phosphodiesterase	Cell cycle, division, growth and fate	downregulated	0,44	0,03	0,24
242	ENSDARP00000124582	cnp	cnp-011	2,,3,-cyclic nucleotide 3, phosphodiesterase	Cell cycle, division, growth and fate	downregulated	0,44	0,03	0,24
243	ENSDARP00000126962	ckbb	ckbb-203	Creatine kinase, brain b	Energy metabolism	downregulated	0,42	0,06	
244	ENSDARP00000073460	crp2	crp2-203	C-reactive protein 2	Immune system	downregulated	0,40	0,21	
245	ENSDARP00000113177	crp	crp-201	C-reactive protein 2	Immune system	downregulated	0,40	0,21	
246	ENSDARP00000072790	vtg2	vtg2-202	Vitellogenin 2	Vitellogenin	downregulated	0,38	<0.001	0,01
247	ENSDARP00000096034	vtg2	vtg2-203	Vitellogenin 2	Vitellogenin	downregulated	0,38	<0.001	0,01
248	ENSDARP00000116297	si:dkey-90l23.2	si:dkey-90l23.2-201	SUEL-TYPE Lectin	Lectins	downregulated	0,35	0,16	
249	ENSDARP00000114321	si:dkey-90l23.2	si:dkey-90l23.2-202	SUEL-TYPE Lectin	Lectins	downregulated	0,35	0,16	
250	ENSDARP00000061164	vtg2	vtg2-201	Vitellogenin 2	Vitellogenin	downregulated	0,26	0,00	0,04
251	ENSDARP00000029296	eef1a1b	eef1a1b-201	Eukaryotic translation elongation factor 1 alpha 1b	Protein synthesis	downregulated	0,23	0,11	
252	ENSDARP00000111742	eef1a1l2	eef1a1l2-202	Eukaryotic translation elongation factor 1 alpha 1, like 2	Protein synthesis	downregulated	0,23	0,11	
253	ENSDARP00000006339	eef1a1l2	eef1a1l2-201	Eukaryotic translation elongation factor 1 alpha 1, like 2	Protein synthesis	downregulated	0,15	0,02	0,20
254	ENSDARP00000089806	si:ch211-251f6.7	si:ch211-251f6.7-001	Fish egg lectin like precursor	Lectins	downregulated	0,14	0,01	0,14
255	ENSDARP00000113985	si:ch211-251f6.7	si:ch211-251f6.7-201	Fish egg lectin like precursor	Lectins	downregulated	0,14	0,01	0,13
256	ENSDARP00000095894	si:dkey-90l23.2	novel	D-galactoside/L-rhamnose binding SUEL lectin domain	Lectins	downregulated	0,14	0,14	
257	ENSDARP00000089804	zgc:136254	zgc:136254-202	Fish egg lectin like precursor	Lectins	downregulated	0,12	0,01	0,13
258	ENSDARP00000129497	zgc:136254	zgc:136254-201	Fish egg lectin like precursor	Lectins	downregulated	0,12	0,01	0,13
259	ENSDARP00000127005	hspa8b	hspa8b-202	Heat shock protein family A (Hsp70) member 8b	Protein synthesis	downregulated	0,00	0,01	0,13

S2 Table. Functional enrichment analysis of differentially abundant proteins in STRING protein-protein interactions networks.

**A) Downregulated in F3 *vtg2*-mutant embryos (N=83)**	
number of nodes:	39
expected number of edges:	20
number of edges:	100
average node degree:	5.13
avg. local clustering coefficient:	0.59
PPI enrichment p-value:	< 1.0e-16

	**Biological Process (Gene Ontology)**			
GO-term	description	# protein	strength	FDR
GO:0042026	Protein refolding	8 of 20	2.43	3.18E-13
GO:0032889	Regulation of vacuole fusion, non-autophagic	2 of 6	2.35	0.0213
GO:0051085	Chaperone cofactor-dependent protein refolding	8 of 39	2.14	1.58E-11
GO:0034620	Cellular response to unfolded protein	8 of 84	1.81	1.48E-09
GO:0006096	Glycolytic process	4 of 47	1.76	0.00088
GO:0006457	Protein folding	9 of 179	1.53	8.47E-09
GO:0009636	Response to toxic substance	4 of 161	1.23	0.033
GO:0006091	Generation of precursor metabolites and energy	6 of 287	1.15	0.0023
GO:0009117	Nucleotide metabolic process	6 of 449	0.96	0.0196
GO:0005975	Carbohydrate metabolic process	6 of 495	0.91	0.0299
GO:0033554	Cellular response to stress	10 of 1449	0.67	0.0149
GO:0006950	Response to stress	13 of 2723	0.51	0.0308

	**Molecular Function (Gene Ontology)**			
GO-term	description	# protein	strength	FDR
GO:0004113	2,3-cyclic-nucleotide 3-phosphodiesterase activity	2 of 2	2.83	0.0031
GO:0045735	Nutrient reservoir activity	5 of 7	2.68	4.03E-09
GO:0102751	Glucosyltransferase activity	2 of 3	2.65	0.0047
GO:0008466	Glycogenin glucosyltransferase activity	2 of 3	2.65	0.0047
GO:0098973	Structural constituent of postsynaptic actin cytosk.	2 of 5	2.43	0.008
GO:0004634	Phosphopyruvate hydratase activity	2 of 5	2.43	0.008
GO:0044183	Protein folding chaperone	8 of 22	2.39	1.71E-13
GO:0051787	Misfolded protein binding	8 of 25	2.34	2.02E-13
GO:0030169	Low-density lipoprotein particle binding	2 of 13	2.02	0.0339
GO:0001848	Complement binding	2 of 16	1.93	0.0341
GO:0051082	Unfolded protein binding	8 of 110	1.69	4.87E-09
GO:0031072	Heat shock protein binding	8 of 112	1.68	4.87E-09
GO:0005319	Lipid transporter activity	6 of 146	1.44	3.75E-05
GO:0016835	Carbon-oxygen lyase activity	3 of 77	1.42	0.0339
GO:0016887	ATPase activity	9 of 504	1.08	2.19E-05
GO:0031625	Ubiquitin protein ligase binding	4 of 248	1.04	0.0478
GO:0017111	Nucleoside-triphosphatase activity	10 of 997	0.83	0.00051
GO:0005524	ATP binding	12 of 2547	0.5	0.0339
GO:0035639	Purine ribonucleoside triphosphate binding	13 of 3139	0.45	0.0393
GO:0032555	Purine ribonucleotide binding	13 of 3215	0.44	0.0478
GO:0000166	Nucleotide binding	14 of 3490	0.43	0.0339
GO:0003824	Catalytic activity	24 of 7610	0.33	0.0047

	**Cellular Component (Gene Ontology)**			
GO-term	description	# protein	strength	FDR
GO:0097433	Dense body	2 of 3	2.65	0.0305
GO:0000015	Phosphopyruvate hydratase complex	2 of 5	2.43	0.0305
GO:0005576	Extracellular region	12 of 2383	0.53	0.0406
GO:0005829	Cytosol	15 of 3167	0.51	0.0305

	KEGG Pathways			
pathway	description	# protein	strength	FDR
dre00500	Starch and sucrose metabolism	2 of 36	1.58	0.0267
dre00010	Glycolysis / Gluconeogenesis	4 of 74	1.56	0.00015
dre03040	Spliceosome	7 of 133	1.55	1.11E-07
dre01230	Biosynthesis of amino acids	4 of 83	1.51	0.0002
dre04141	Protein processing in endoplasmic reticulum	9 of 191	1.5	1.95E-09
dre01200	Carbon metabolism	5 of 127	1.43	5.15E-05
dre04144	Endocytosis	7 of 302	1.2	1.72E-05
dre05132	Salmonella infection	5 of 251	1.13	0.00075
dre04010	MAPK signaling pathway	7 of 373	1.1	5.15E-05
dre01100	Metabolic pathways	8 of 1672	0.51	0.0454

	Subcellular localization (COMPARTMENTS)			
compartment	description	# protein	strength	FDR
GOCC:0042718	Yolk granule	5 of 7	2.68	7.57E-09
GOCC:0060417	Yolk	5 of 8	2.63	7.57E-09
GOCC:0000015	Phosphopyruvate hydratase complex	2 of 5	2.43	0.01
GOCC:0043202	Lysosomal lumen	2 of 12	2.05	0.0382
GOCC:0043209	Myelin sheath	2 of 13	2.02	0.0397
GOCC:0034362	Low-density lipoprotein particle	2 of 14	1.99	0.0412
GOCC:0005577	Fibrinogen complex	2 of 14	1.99	0.0412
GOCC:0005833	Hemoglobin complex	2 of 17	1.9	0.046
GOCC:0031983	Vesicle lumen	5 of 62	1.74	1.45E-05
GOCC:0043233	Organelle lumen	18 of 1946	0.8	3.59E-08
GOCC:0031974	Membrane-enclosed lumen	18 of 1946	0.8	3.59E-08
GOCC:0070013	Intracellular organelle lumen	13 of 1936	0.66	0.00061
GOCC:0005737	Cytoplasm	32 of 8863	0.39	2.42E-07
GOCC:0043227	Membrane-bounded organelle	26 of 10040	0.24	0.0412

B) Upregulated in F3 *vtg2*-mutant embryos (N=176)	
number of nodes:	138
expected number of edges:	123
number of edges:	270
average node degree:	3.91
avg. local clustering coefficient:	0.534
PPI enrichment p-value:	< 1.0e-16

	Biological Process (Gene Ontology)			
GO-term	description	# protein	strength	FDR
GO:0090131	Mesenchyme migration	7 of 7	2.28	2.11E-10
GO:0046314	Phosphocreatine biosynthetic process	4 of 7	2.04	0.00011
GO:0006729	Tetrahydrobiopterin biosynthetic process	4 of 13	1.77	0.00059
GO:0006559	L-phenylalanine catabolic process	4 of 14	1.74	0.0007
GO:0006334	Nucleosome assembly	33 of 142	1.65	1.83E-38
GO:0007338	Single fertilization	8 of 88	1.24	2.19E-05
GO:0051276	Chromosome organization	34 of 1063	0.79	1.79E-14
GO:0043933	Protein-containing complex subunit organization	34 of 1270	0.71	2.59E-12
GO:0007186	G protein-coupled receptor signaling pathway	23 of 1196	0.57	4.19E-05
GO:0022607	Cellular component assembly	36 of 2261	0.48	8.08E-07
GO:0006996	Organelle organization	42 of 3539	0.36	9.01E-05
GO:0007166	Cell surface receptor signaling pathway	23 of 1931	0.36	0.0449
GO:0009987	Cellular process	122 of 19185	0.09	0.0018

	Molecular Function (Gene Ontology)			
GO-term	description	# protein	strength	FDR
GO:0004459	L-lactate dehydrogenase activity	3 of 5	2.06	0.0016
GO:0032190	Acrosin binding	8 of 14	2.04	9.39E-11
GO:0004155	6,7-dihydropteridine reductase activity	4 of 7	2.04	9.24E-05
GO:0070404	NADH binding	4 of 8	1.98	0.00012
GO:0031386	Protein tag	4 of 8	1.98	0.00012
GO:0004111	Creatine kinase activity	4 of 8	1.98	0.00012

GO:0045735	Nutrient reservoir activity	3 of 7	1.91	0.0029
GO:0070402	NADPH binding	4 of 15	1.71	0.00061
GO:0046982	Protein heterodimerization activity	33 of 339	1.27	9.10E-28
GO:0005200	Structural constituent of cytoskeleton	6 of 75	1.19	0.00092
GO:0030246	Carbohydrate binding	30 of 454	1.1	1.25E-20
GO:0046983	Protein dimerization activity	36 of 1037	0.82	1.36E-16
GO:0004930	G protein-coupled receptor activity	20 of 899	0.63	3.05E-05
GO:0003677	DNA binding	34 of 3347	0.29	0.0141
GO:1901363	Heterocyclic compound binding	62 of 8068	0.17	0.0344
GO:0097159	Organic cyclic compound binding	62 of 8150	0.16	0.0419
GO:0005488	Binding	121 of 17458	0.12	3.54E-06

	**Cellular Component (Gene Ontology)**			
GO-term	description	# protein	strength	FDR
GO:0035805	Egg coat	8 of 15	2.01	4.78E-11
GO:0000786	Nucleosome	32 of 157	1.59	2.11E-36
GO:0043232	Intracellular non-membrane-bounded organelle	51 of 4406	0.35	1.72E-06

	**KEGG Pathways**			
pathway	description	# protein	strength	FDR
dre00270	Cysteine and methionine metabolism	4 of 51	1.18	0.0139
dre00620	Pyruvate metabolism	3 of 41	1.15	0.0289
dre00330	Arginine and proline metabolism	4 of 63	1.08	0.0139
dre00010	Glycolysis / Gluconeogenesis	4 of 74	1.01	0.0164
dre04540	Gap junction	6 of 122	0.97	0.0095
dre04260	Cardiac muscle contraction	5 of 129	0.87	0.0164
dre04145	Phagosome	6 of 163	0.85	0.0139
dre04210	Apoptosis	6 of 181	0.8	0.0139
dre04530	Tight junction	7 of 215	0.79	0.0139

	**Subcellular localization (COMPARTMENTS)**			
compartment	description	# protein	strength	FDR
GOCC:0035805	Egg coat	8 of 14	2.04	2.94E-11
GOCC:0000786	Nucleosome	32 of 63	1.99	4.57E-47
GOCC:0044815	DNA packaging complex	32 of 72	1.93	7.12E-46
GOCC:0042718	Yolk granule	3 of 7	1.91	0.0024
GOCC:0060417	Yolk	3 of 8	1.86	0.0031
GOCC:0030312	External encapsulating structure	8 of 33	1.67	7.18E-09
GOCC:0032993	protein-DNA complex	32 of 133	1.66	8.12E-39
GOCC:0000785	Chromatin	33 of 479	1.12	4.69E-24
GOCC:0031983	Vesicle lumen	4 of 62	1.09	0.0472
GOCC:0005694	Chromosome	34 of 935	0.84	1.61E-16
GOCC:0030054	Cell junction	25 of 1351	0.55	6.75E-06
GOCC:0043232	Intracellular non-membrane-bounded organelle	55 of 3173	0.52	3.92E-14
GOCC:0043228	Non-membrane-bounded organelle	55 of 3190	0.52	4.24E-14
GOCC:0032991	Protein-containing complex	45 of 5262	0.21	0.0408
GOCC:0110165	Cellular anatomical entity	131 of 20534	0.09	4.66E-06

S3 Table. Targets and primers utilized in this study. Target oligo and screening primer names are given according to **Fig 1**. CRISPR recognition NGG motifs are highlighted by bold typeface on sequences. Position of primers and target sites on vitellogenin (Vtg) yolk protein (YP) domains are given on the far right columns.

**Target Oligos**	**Sequence**	**Vtg YP domain**
sg21_Rv	**GGC**AGGAAAAACTTGATCCT	LvH
sg22_Rv	**GGA**ACGGTTCTCTCAGCAGA	LvH
sg23_Fw	**GGA**CAGACTTGTGGACTTTG	Bc
**Screening Primers**	**Sequence**	**Vtg YP domain**
21_Fw	ACATTTCCCAACGAGGGTGT	LvH (intron)
21_Rv	TGAGCTCCAGCCTTGAAGAA	LvH (intron)
22_Fw	CCATCCTCAATCTCGTTCCAG	LvH (intron)
22_Rv	ACAGCCAGAATGACAAAATTAGTTG	LvH (intron)
23_Fw	CAGCAGCCTTCCTTATCAG	Bc
23_Rv	ACAGGCTCAACGGAATAGCA	Ct
**qPCR Primers**	**Sequence**	**Vtg YP domain**
vtg2_Fw	TGCCGCATGAAACTTGAATCT	Ct
vtg2_Rv	GTTCTTACTGGTGCACAGCC	
rpl13a_Fw	TCTGGAGGACTGTAAGAGGTATGC	N/A
rpl13a_Rv	AGACGCACAATCTTGAGAGCAG	
eif1a_Fw	CTGGAGGCCAGCTCAAACAT	N/A
eif1a_Rv	ATCAAGAAGAGTAGTACCGCTAGCATTAC	
