# Supplementary figures and images for: Genomic disturbance of vitellogenin 2 (vtg2) leads to vitellin membrane deficiencies and significant mortalities at early stages of embryonic development in zebrafish (Danio rerio)
Source: Sci Rep. 2023 Nov 1;13:18795. doi: 10.1038/s41598-023-46148-2 (PMC10620220; doi:10.1038/s41598-023-46148-2)

Fig 1C. original images

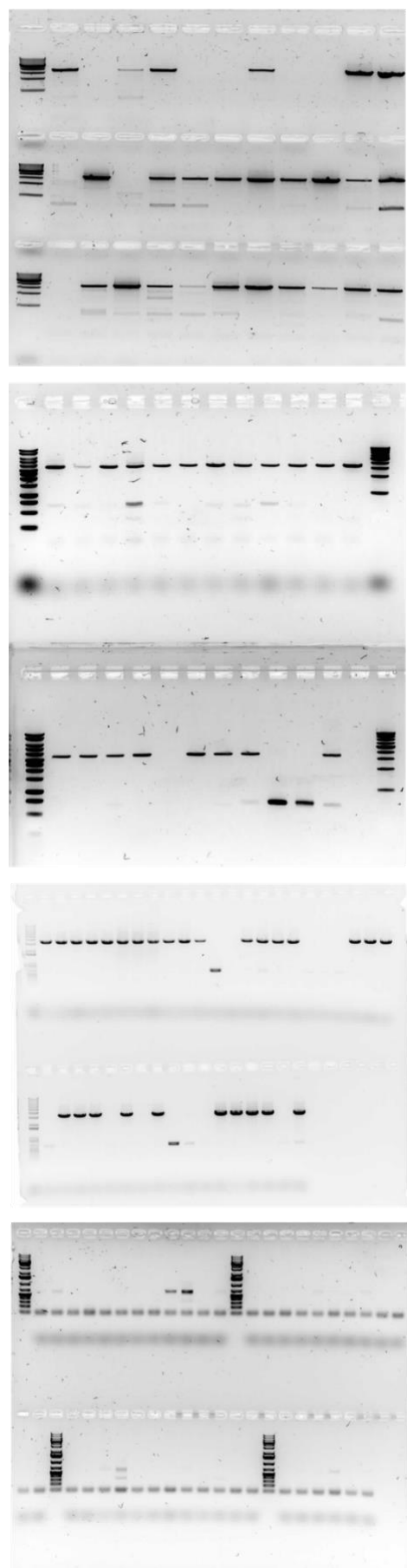

Fig 7. Original images

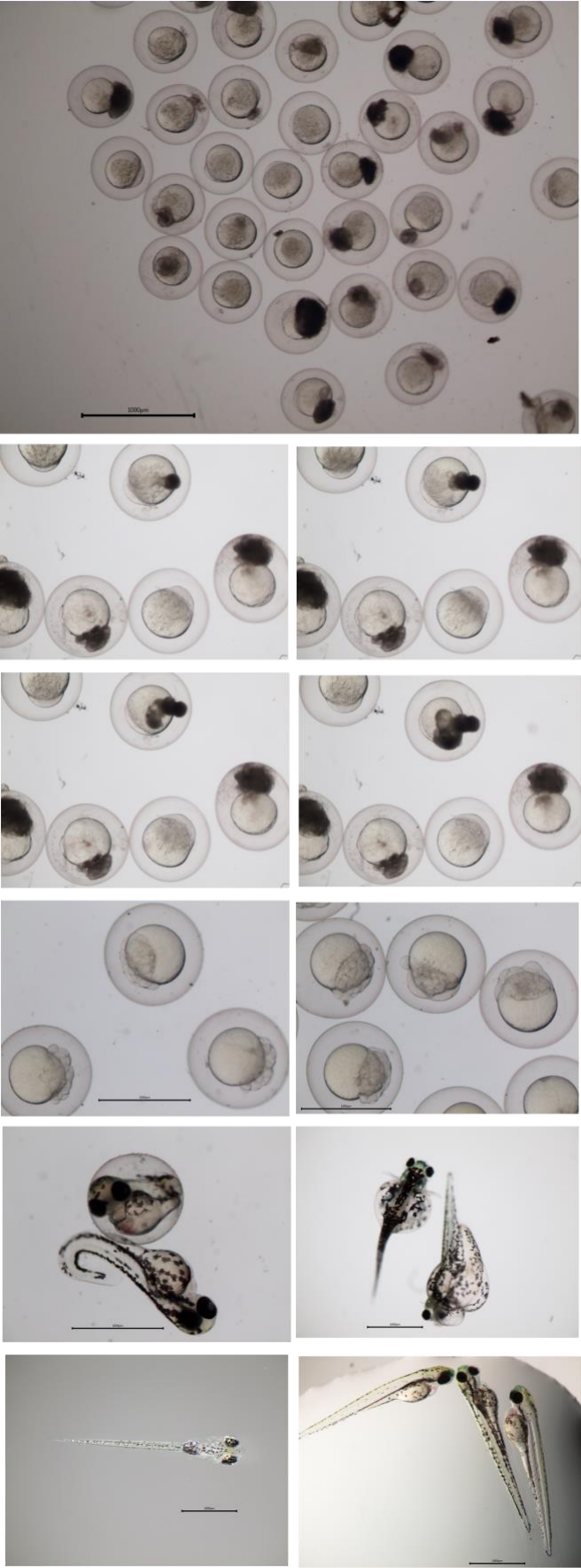

Supplement: Supplementary file 2 — Supplementary Information 2. [file 41598_2023_46148_MOESM2_ESM.pdf]
